# *In situ* formation of low molecular weight organogelators for slick solidification†

**DOI:** 10.1039/c9ra10122e

**Published:** 2020-04-01

**Authors:** Jean-Marie R. Peron, Hollie Packman, William J. Peveler, Joseph C. Bear

**Affiliations:** Department of Chemical and Pharmaceutical Sciences, Kingston University Kingston upon Thames Surrey KT1 2EE UK j.bear@kingston.ac.uk; Department of Earth Science and Engineering, South Kensington Campus, Imperial College London SW7 2AZ UK; School of Chemistry, Joseph Black Building, University of Glasgow Glasgow G12 8QQ UK

## Abstract

We have investigated the *in situ* formation of Low Molecular Weight Organogelator (LMWO) molecules in oil-on-water slicks through dual reactive precursor injection. This method alleviates the need for any carrier solvent or prior heating, therefore reducing the environmental impact of LMWOs, giving instantaneous gelation, even at low temperatures (−5 °C). We show minimal leaching from our gels into the water layer.

## Abstract 2

Instantaneous gelation: a reactive precursors approach for the near-instant gelation of oil-on-water slicks down to −5 °C.

## Graphical abstract

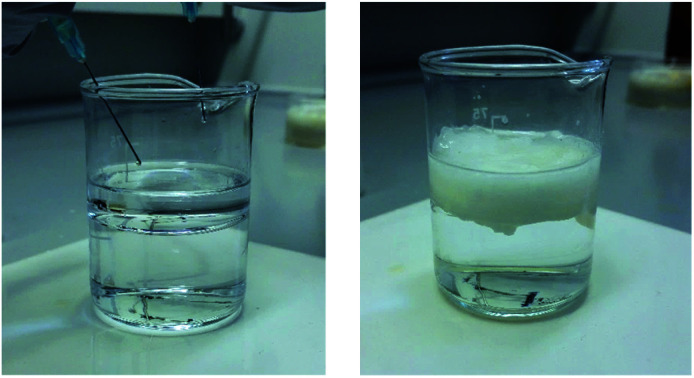

Low molecular weight organogelators (LMWOs or LMOGs) are small molecules designed to form supramolecular networks on addition to oil, turning the oil into a solid gel.^1–5^ Once gelled, the oil can then be more easily removed. This makes LMWOs of great interest in the clean-up of marine oil and fuel spills, especially close to the shoreline or on bodies of inland water. A key advantage of LMWOs is that their properties can be designed to some extent at a molecular level^4^ insofar as one must ensure that the molecule will be able to form a supramolecular network with itself as well as ensuring solubility in the oil. Fig. 1(a) and (b) show the urea-based LMWOs used in this work, with hydrogen bonds between urea groups and π–π stacking between aromatic groups as examples of gelling intermolecular forces shown in Fig. 1(c). The lipophilic part of the LMWO grants the molecule solubility in oil as typically, the gelling intermolecular forces are polar in nature. Therefore, the synthesis of LMWOs often relies on balancing solubility in oil with polar intermolecular forces; too much hydrogen bonding, the compound will not be easily soluble, too little, and the compound simply will not gel.

**Fig. 1:**
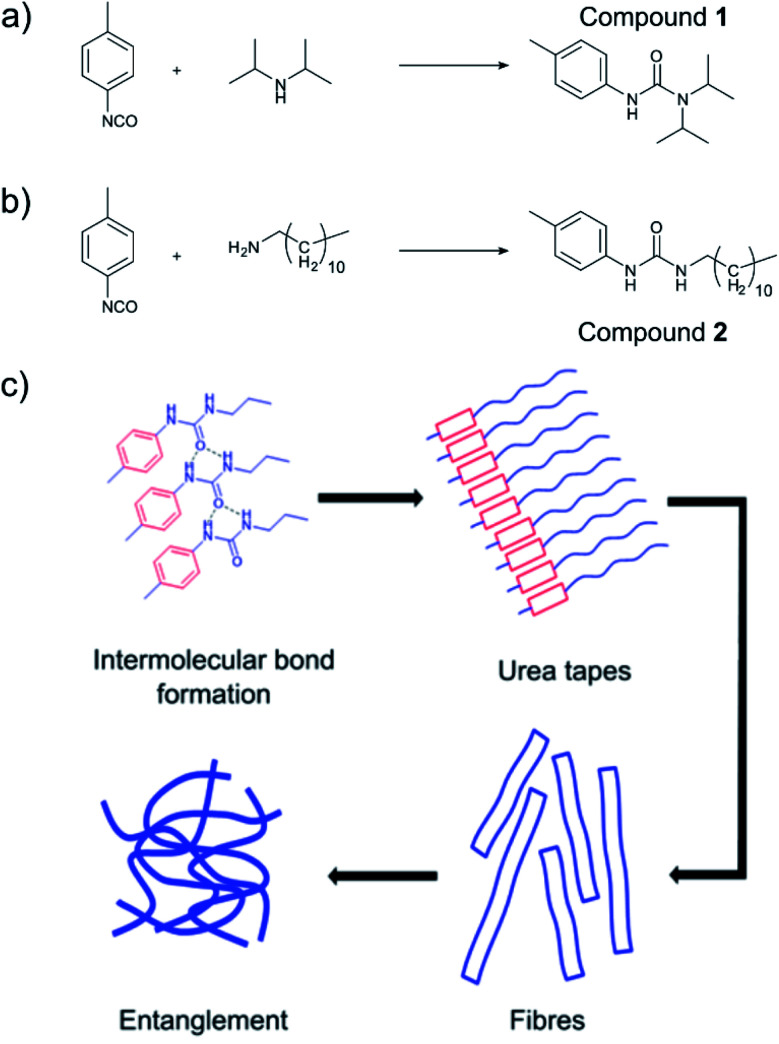
(a) Reaction scheme of *p*-tolyl isocyanate “core” with a diisopropylamine “tail” forming *N*′-(4-methylphenyl)-*N*,*N*-dipropan-2-ylurea (referred to as compound 1), (b) reaction of *p*-tolyl isocyanate with dodecylamine forming *N*-dodecyl-*N*′-(4-methylphenyl)-urea (referred to as compound 2) (c) a scheme demonstrating the hypothetical self-assembly of urea-based LMWOs into supramolecular networks.

Tolyl-isocyanates (“core” groups) are attractive precursors for urea based LMWOs, due to their ready availability from widespread use in poly(urethane) manufacture. They produce urea/urethane moieties on reaction with a nucleophile such as an amine or an alcohol, which readily hydrogen bond, giving the gelator the required intermolecular forces to form the supramolecular network in oils. These urea-based LMWOs have been widely characterised and explored.^1–4^ The choice of nucleophilic “tail” (amine) is critical in order to impart solubility to the LMWO whilst still allowing the self-assembled structure to form. Previously, we have demonstrated a selection of *p*, *m* and *o*-tolyl isocyanates forming stable oil binding gels in sea water.^9^ In this study we examined the properties of a diisopropylamine/*p*-tolyl isocyanate system (Fig. 1(a)), and a dodecylamine/*p*-tolyl isocyanate system (Fig. 1(b)) with an eye to *in situ* application of the gel to an oil spill.

It is the delivery of the LMWO to the hydrocarbon slick and gelation of the spill that remains a key challenge in environmental oil-spill remediation with LMWOs. The application of the LMWO to the oil has proven to be highly exacting simply due to the strength of the gelling intermolecular forces, and a successful LMWO will most commonly manifest itself as a solid. Therefore, in order to solubilise a solid LMWO in oil, energy (heat) has to be applied to the system to overcome these intermolecular forces and force dissolution. On mixing with the oil and cooling, the intermolecular forces can re-form and the oil will gel.

This is a severe limitation of LMWOs in the oil-spill clean-up role, as they must either: (a) be applied hot, increasing deployment difficulty and energetic cost or (b) to hasten dissolution in oil, they would have to be deployed in a carrier solvent, increasing potential environmental consequences. Potentially, this is also a reason why LMWOs would only be suited to inshore clean-up as the cooling on aerial deployment would mean the LMWO would solidify before reaching the oil, preventing dissolution and gelation.

Several groups have published investigations along these lines, such as: using heated solutions of LMWOs,^5,6^ or supergelators dissolved in flammable ethanol/ethyl acetate blends of solvents to aid gel dissolution.^7–9^ Sureshan *et al.* reported alkyl 4,6-*O*-benzylidene-glucopyranoside derivatives which can be applied as a powder and will gel oil mixtures on seawater^5^ and more recently, Zhang *et al.* reported d-gluconic acetal-based powder gelators able to gel oil slicks at room temperature.^10^

Another emerging school of thought involved the combinatorial approach of LMWOs coupled with sorbents.^11^ For example, the use of a supergelator (definition: a critical gelation concentration (CGC) of <0.1 wt%)^12^ contained within a cellulose pulp matrix has been shown to be very effective at absorbing oil and affecting the release of the gelator into the oil.^13^ This approach alleviates the need for a carrier solvent and solves the problem of dissolution, but does require the presence of a solid matrix to work.

In an alternative solution to the aforementioned challenges, we utilised the rapid reaction of isocyanate and amine to form a LMWO *in situ*, a method first utilised by Suzuki *et al.*, who used the *in situ* synthesis of urea-based LMWOs to gel a variety of solvents in 2004. Here we extend the method to oil-on-sea water slicks.^14^ We also explore for the first time, the influence of temperature on *in situ* gelation, going below room temperature to more accurately simulate oceanic conditions. Herein we can report the successful, rapid gelation of 1-octadecene using compound 1 as a slick on cold seawater (−5 °C), an experiment essential for validating this method in cold environments.

If two liquid precursors, such as diisopropylamine and *p*-tolyl isocyanate were sprayed into an oil in close proximity or one after another, a gel can form in the oil *in situ*, thus alleviating the need for elevated temperature or a carrier solvent. We examined this hypothesis by simulating an oil slick utilising 1-octadecene on deionised water, before rapid injection of equimolar amounts of *p*-tolyl isocyanate and amine (diisopropylamine (forming *N*′-(4-methylphenyl)-*N*,*N*-dipropan-2-ylurea), henceforth referred to as compound 1 or dodecylamine (forming *N*-dodecyl-*N*′-(4-methylphenyl)-urea), henceforth referred to as compound 2). On injection, either the isocyanate after the amine or *vice versa* (or indeed simultaneously), urea fibres began to form rapidly, completely gelling the 1-octadecene within 60 seconds (Fig. 2, 3 and ESI Video†), even on slicks at low temperature on seawater at −5 °C (see Fig. S2†). Rapid gelation within 60 seconds occurred with LMWO concentrations down to 2 wt% with the diisopropylamine/*p*-tolyl isocyanate system (1), and 5 wt% dodecylamine/*p*-tolyl isocyanate system (2), below which the reaction was slower with weaker gel consistency, and did not survive the “inversion test” (Fig. S1†). We successfully managed to form an LMWO *in situ* in an oil on water slick, through rapid reaction of isocyanate and amine precursors, as shown by nuclear magnetic resonance (NMR) spectroscopy in the ESI.† Indeed, water should compete for reaction with the isocyanate, but the localised high concentration precluded this. The NMR of the resulting gels did not show evidence of the isocyanate reaction with water in the oil slick, and no sequestered water in the oil. The resultant ureas and gelators were isolable from oil and could be extracted by distillation or centrifugation, as demonstrated in our previous work.^4^ We successfully repeated these experiments with kerosene and motor oil and several other oils at −5 °C (details in Table S1 ESI†).

**Fig. 2:**
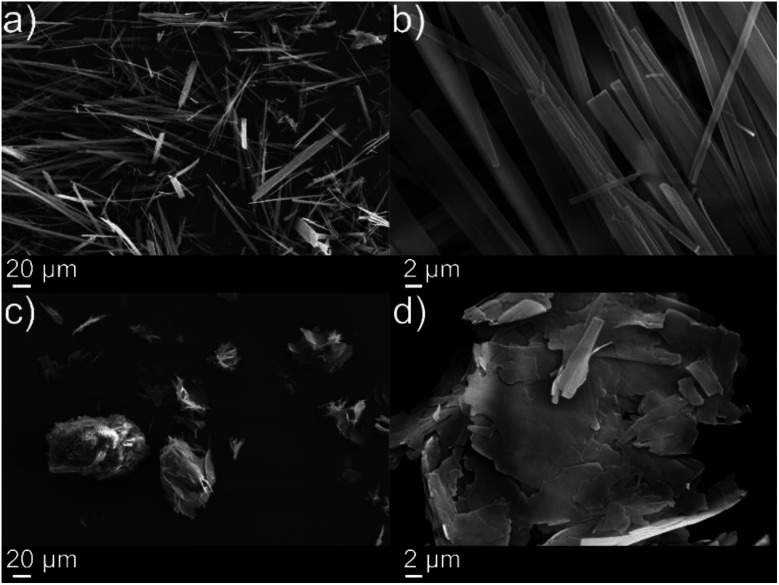
Scanning electron microscope images of xerogels of: (a) and (b) 10 wt% diisopropylamine/*p*-tolyl isocyanate system (compound 1), and (c) and (d) 10 wt% dodecylamine/*p*-tolyl isocyanate system (compound 2).

**Fig. 3:**
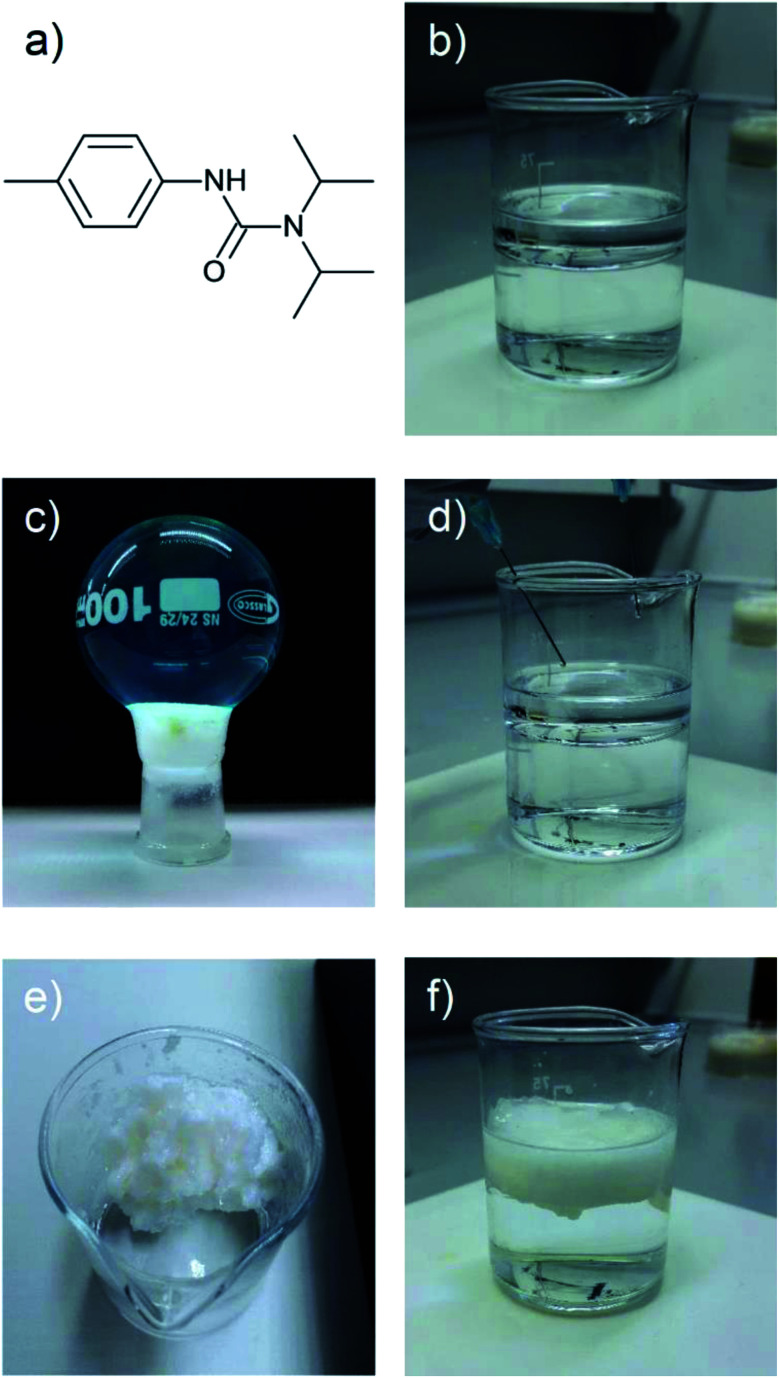
Photographs demonstrating the experimental protocol. (a) is gelator 1, the product from the reaction *p*-tolyl isocyanate with diisopropylamine, (b), (d) and (f) show the process of simultaneous injection of *p*-tolyl isocyanate and diisopropylamine into a 1-octadecene on water slick forming a gel in (f), (c) a 100 ml round bottom flask filled with water held back by a 1/1-octadecene gel and (e) illustrates complete separation of a 1/1-octadecene gel and water.

The selected “cores” and “tails” are low molecular weight, with neither exceeding 320 g mol^−1^. Precursors are all inexpensive (*p*-tolyl isocyanate is a poly(urethane) precursor) and alkyl amines which are readily available and inexpensive. The synthesis of ureas/carbamate from isocyanates and primary amines/alcohols is an instantaneous, facile one-pot reaction giving a pure product in high yield.^4^ Purification involved removal of the solvent (if any) and drying *in vacuo*. The process can easily be adapted to the kilogram scale, with *prima facae* evidence being the poly(urethane) industry. The LMWOs formed by this reaction are of unknown toxicity, but neither exhibited significant solubility in water, so are unlikely to pose a long-term threat to aquatic life.

The *in situ* reaction was also very effective at gelling thin oil slicks (*ca.* 2 mm) and facilitated the collection of oil as evidenced in Fig. S3† and 3e respectively. SEM images in Fig. 2 (xerogels of dodecylamine/*p*-tolyl isocyanate and diisopropylamine/*p*-tolyl isocyanate formed from gelling cyclohexane) demonstrate the formation of urea “tapes” in the same fashion as our previous work.^4^ The dodecylamine/*p*-tolyl isocyanate gel however formed more plate-like structures (Fig. 2(c) and (d)).

The rapidity of reaction is very important for deployment of the *in situ* method system for fast clean-up of oil spills. Indeed, the use of diisopropylamine over dodecylamine facilitates a more rapid reaction, as evidenced by Video S2 in the ESI.† We postulate that this is simply due to the small size of the diisopropylamine *versus* the dodecylamine, with the smaller diisopropylamine being able to react faster. Further to this, pump oil on river water (obtained from the River Thames near Kingston) was gelled successfully with both the dodecylamine/*p*-tolyl isocyanate and diisopropylamine/*p*-tolyl isocyanate systems. In terms of concentrations of precursors applied, we were able to invert 1 ml of 1-octadecene gels formed with the precursors applied against 2 ml of deionised water (see Fig. S4†). The integrity of gels of 1 was maintained down to 1 wt%, whereas for 2, integrity was maintained down to 2 wt% of gelator.

NMR spectroscopy confirmed that both gelators 1 and 2 were formed in high yield on reaction of *p*-tolyl isocyanate and diisopropylamine/dodecylamine respectively, in the absence of solvent (ESI†). Furthermore, the same reagents reacted together successfully and in quantitative yields when added separately or together to a 1-octadecene layer on an aqueous layer. The gel formed in those cases dissolved fully in CDCl_3_ with no significant amount of water present in the gel. This seemed to indicate no sequestration of water by the gel as it formed.

One of the main problems with the approach outlined herein is toxicity. The safety datasheets (SDS) for both diisopropylamine and *p*-tolylisocyanate class them as irritants and as toxic. In order to ascertain the amount of precursors and gelator that leached into the aqueous phase, various slicks of 1-octadecene were created on seawater and river water before injection of precursors into the oil layer. The aqueous phases were then analysed for leachates by NMR. The results are summarised in Table 1.

**Table 1:** Amounts of leachates (*p*-tolylamine, diisopropylamine, dodecylamine, gelator) found in aqueous layers

Leaching experiment	Respective leachate (parts per million)			
	*p*-Tolylamine^a^	Diisopropyl-amine	Dodecylamine	Gelator
1 on sea water	30	27 (S/N = 58)	—	50
1 on river water	35	28	—	61
1 2% on river water^b^	33	51	—	40
2 on sea water	26	—	ND	ND
2 on river water	27	—	ND	ND

a Hydrolysis product of *p*-tolylisocyanate, internal standard was dimethylsulfone, *δ*_H_ (D_2_O) = 3.15 ppm (internal reference TSP), ND = not detectable above noise level.

b Sample from 2% wt/wt gelator : oil ratio (*i.e.* 2 tenths of previous sample). The values reported above are those determined from a single NMR sample on one peak for each analyte where a signal to noise ratio (S/N) above 240 was measured (unless otherwise noted). When other peaks were available for integration which had S/N in as low as 77 the resulting concentrations were essentially the same as those presented above.

The leaching experiments seem to indicate very little dodecylamine or its gelator compound 2 leached from the oil (1-octadecene) to the aqueous layer. However, a trace amount of diisopropylamine was detected by ^1^H NMR in the case of 1, as was *p*-tolylamine the by-product resulting from the hydrolysis of isocyanate. These findings indicate that the long alkyl chain in dodecylamine and 2 prevent appreciable leaching into aqueous media. The leaching of *p*-tolylisocyanate was consistent in both river and seawater. This led the investigators to conclude that if a more hydrophobic isocyanate was used as a LMWO precursor in conjunction with dodecylamine, then leaching could be prevented altogether. Therefore, we have largely overcome the toxicity hazards associated with the *in situ* delivery method, paving the way for similar *in situ* generation of LMWO molecules from reactive precursors in future. We note that recent advances in “green isocyanate” synthesis from renewable feedstocks may also reduce environmental concerns of our method.^15,16^

## Experimental

### Materials

*p*-Tolyl isocyanate (99%), dodecylamine (98%) and 1-octadecene (technical grade, 90%) were purchased from Sigma Aldrich Ltd. and used as received. Diisopropylamine (99%) was purchased from ACROS and used as received. UHQ deionised water with a resistivity of not less than 18.2 MΩ cm^−1^ was used. Seawater from Brighton, UK, was provided by Dr Rosa Busquets (Kingston University), and was used as received. River water was directly sourced from the River Thames, in Kingston upon Thames, UK, and was used without further treatment.

### Instrumentation

NMR spectra were acquired on a Bruker Avance III 400 MHz (^1^H) FT-NMR spectrometer equipped with a 5 mm room temperature probehead (PABBO BB-1H/D Z-GRD, broadband multinuclear, autotune) from Bruker BioSpin GmbH, Switzerland, and controlled with TopSpin 3.5.7 and Icon NMR 5.0.7 © 2017 Bruker Biospin GmbH. Samples (*ca.* 30 mg dry gelator or 100 mg gelator + oil) were prepared in CDCl_3_ (Goss Scientific Instruments Ltd., Crewe, UK) and spiked with a trace amount of tetramethylsilane (Merk, Gillingham, UK) as the 0 ppm internal reference for ^1^H spectra. 1D (^1^H, ^13^C) and 2D (^1^H–^1^H COSY, ^1^H–^13^C HSQC) NMR experiments were carried out to confirm the presence of gelator 1, and/or starting reagents. Scanning electron microscope images were taken on a Zeiss EVO 50 SEM at an acceleration voltage of 10 kV.

### Methods

For the *in situ* gelation studies, unless otherwise stated, 1-octadecene (40 ml) on deionised water (200 ml) were used to simulate oil slicks at room temperature. Equimolar amounts (depending on gelator concentration in oil required) of *p*-tolyl isocyanate and diisopropylamine or dodecylamine were rapidly injected into the 1-octadecene (top) layer, rapidly reacting and forming the urea LMWO *in situ*, which in turn gelled the oil (see Video in the ESI†). A salt-ice bath was used to cool seawater (*ca.* 90 ml) and 1-octadecene (*ca.* 5 ml) to −5 °C before addition of isocyanate and amine (results can be seen in Fig. S2†). The NMR spectra and further experimental details are provided in the ESI.† The NMR results are summarised below.

#### For *N*′-(4-methylphenyl)-*N*,*N*-dipropan-2-ylurea (compound 1)

^1^H NMR (400 MHz, CDCl_3_) *δ* 7.25(2H, d, *J* = 8.0 Hz, 2- and 6-H), 7.08 (2H, d, *J* = 8.0 Hz, 3- and 5-H), 6.13 (1H, s, **NH**), 3.97 (2H, sept, *J* = 6.9 Hz, 2 × CH(**CH**_**3**_)_2_), 1.31 (12H, d, *J* = 6.9 Hz, 2 × **CH**(CH_3_)_2_); ^13^C NMR (100 MHz, CDCl_3_) *δ* 154.80 (C=O), 136.77 (C_Q_), 132.10 (C_Q_), 129.34 (5-CH), 119.89 (6-CH), 45.5 (**CH**(CH_3_)_2_), 21.7 (CH(**CH**_**3**_)_2_), 20.70 (4-CH_3_).

#### For *N*-dodecyl-*N*′-(4-methylphenyl)-urea (compound 2)

^1^H NMR (600 MHz, DMSO-d_6_) *δ* 8.19 (1H, s, Ph**NH**CONHCH_2_–) 7.24 (2H, d, *J* = 8.3 Hz, 2- and 6-H), 7.00 (2H, d, *J* = 8.3 Hz, 3- and 5-H), 6.00 (1H, t, *J* = 5.6 Hz, PhNHCO**NH**CH_2_–), 3.05 (2H, brd dd, *J* = 12.8, 6.8 Hz, 1′-**CH**_**2**_), 1.42–1.38 (2H, m, 2′-CH_2_), 1.29–1.21 (18H, m, –**(CH**_**2**_**)**_**9**_CH_3_)_,_0.85 (3H, t, *J* = 7.0 Hz, **CH**_**3**_).

^13^C NMR (150 MHz, DMSO-d_6_) *δ* 155.73 (C=O), 138.52 (1-C_Q_), 130.02 (4-C_Q_), 129.43 (3- and 5-CH), 118.16 (2- and 6-CH), 39.49 (1′-CH_2_), 31.74 (CH_2_), 30.24 (2′-CH_2_), 29.49 (CH_2_), 29.48 (CH_2_), 29.46 (CH_2_), 29.45 (CH_2_), 29.23 (CH_2_), 29.15 (CH_2_), 26.84 (3′-CH_2_), 22.53 (CH_2_), 20.73 (4-CH_3_), 14.38 (12′-CH_3_).

^15^N NMR (60.8 MHz, DMSO-d_6_) 103.60 (Ph**NH**CONHCH_2_–), 86.20 (PhNHCO**NH**CH_2_–) (from projection of f1 of ^1^H–^15^N HSQC).

For the leaching studies, gels were prepared using the same methodology as above, but substituting either river water or seawater for deionised water. The aqueous layers were subjected to NMR analysis as described in the ESI.† Briefly, the aqueous layer below the newly formed gel was gently homogenised and sampled for NMR analysis. Each NMR tube was assembled using the aqueous layer from the gelation experiment (0.540 ml), D_2_O (0.060 ml) and a bolus (0.030 ml) of dimethyl sulfone (Merck, Gillingham, UK) stock solution (9.10 mg in 1.000 ml) as internal standard. Prior to NMR analysis, samples were spiked with a trace amount of 3-(trimethylsilyl)propionic acid-d_4_ sodium salt (TSP) (Merck, Gillingham, UK) as the internal reference (0 ppm).

## Conclusions

Whilst many new supergelators for oil are being designed, produced and tested, it is in the delivery of such LMWOs where work must focus to maximise the societal and environmental benefits. In this paper, we have successfully shown that the rapid reaction between an isocyanate and an amine in a simulated oil spill will successfully and rapidly gel the oil which can then be removed. This method removes the need for a carrier solvent or solid matrix, and we have shown that no significant leachates infiltrate the aqueous phase of a slick, especially when more hydrophobic precursors are used. This makes the *in situ* method a more environmentally friendly approach for oil spill remediation, therefore paving the way for future LMWOs to be designed from reactive precursors. The *in situ* method has also been used to demonstrate the gelling of a slick on cold seawater at −5 °C, a vital property needed for deployment in cold seas, and an advantage over established LMWO delivery methods.

## Conflicts of interest

There are no conflicts to declare.

## Supplementary Material

RA-010-C9RA10122E-s001

RA-010-C9RA10122E-s002

RA-010-C9RA10122E-s003
