# Supplementary material for: In situ formation of low molecular weight organogelators for slick solidification
Source: RSC Adv. 2020 Apr 1;10(23):13369–73. doi: 10.1039/c9ra10122e (PMC9051380; doi:10.1039/c9ra10122e)
Supplement: RA-010-C9RA10122E-s001 [file RA-010-C9RA10122E-s001.pdf]

Electronic Supplementary Material (ESI) for RSC Advances.
This journal is © The Royal Society of Chemistry 2020

In-situ Formation of Low Molecular Weight Organogelators for Slick Solidification – Supporting Information

Jean-Marie Peron[a] , Hollie Packman[b] ,William J. Peveler[c] and Joseph C. Bear*[a]

[a] Department of Chemical and Pharmaceutical Sciences, Faculty of SEC, Kingston University, Kingston upon Thames, Surrey, KT1 2EE, UK. Email: j.bear@kingston.ac.uk

[b] Department of Earth Science and Engineering, South Kensington Campus, Imperial College, London, SW7 2AZ, UK.

[c] School of Chemistry, Joseph Black Building, University of Glasgow, Glasgow G12 8QQ, United Kingdom.

Table of Contents

Table of Contents 2

Figure S1: 3

Figure S2 4

Figure S3 5

Figure S4 6

Figure S5 7

Part I: Preparation of 7B in situ and analysis of resulting gel 8

The NMR spectroscopy 8

Instrumentation 8

Sample preparation 8

NMR analysis 8

Part II: Preparation and analysis of 2 14

NMR spectroscopy 14

Instrumentation 14

Sample preparation 15

NMR analysis 15

NMR of gelator 2 after removal of n-hexane under vacuum 16

Part III: Aqueous leaching experiments 21

Instrumentation 21

Sample preparation 21

NMR analysis 21

References 23

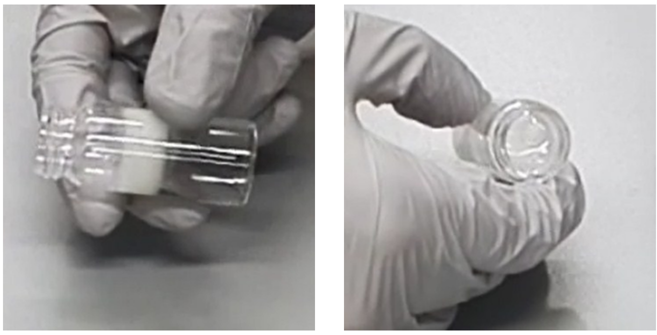

Figure S1: Inversion tests for 1-octadecene slicks on deionised water of: left: **1** and right: **2**. Note that at 5 wt.% gelator concentration, with a larger volume of 1-octadecene (not seen with conditions described in Figure S4) , the integrity of **2** gel was compromised in the inversion test.

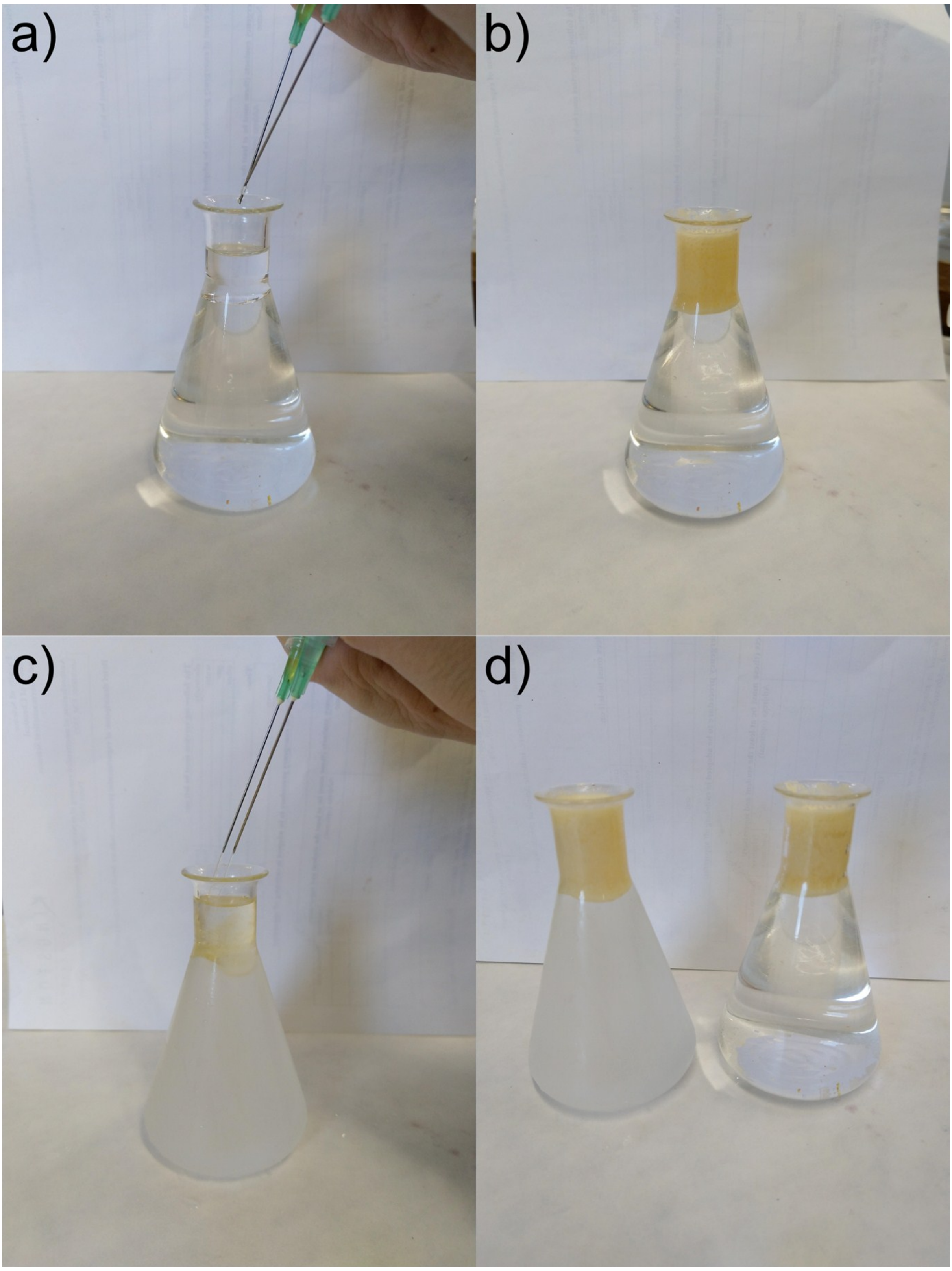

Figure S2: Photographs showing Compound **1** gelling a 1-octadecene slick at room temperature (a) and b)) and at -5 °C (c)). 1-octadecene slicks were placed above seawater and the two flasks are shown for comparison in d).

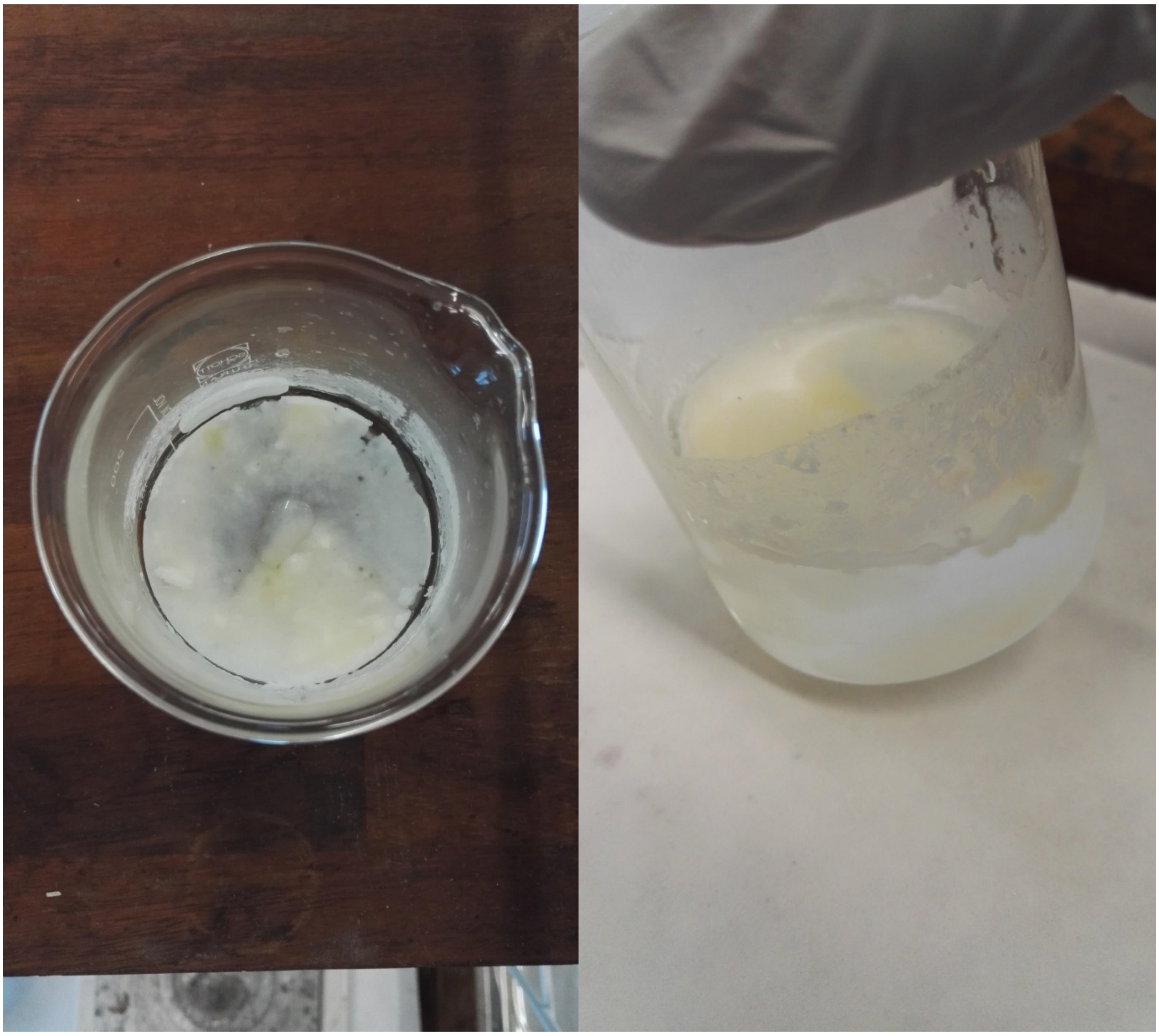

Figure S3: Photograph showing Compound **1** gelling thin 1-octadecene slicks (*ca.* 2 mm thick) on deionised water.

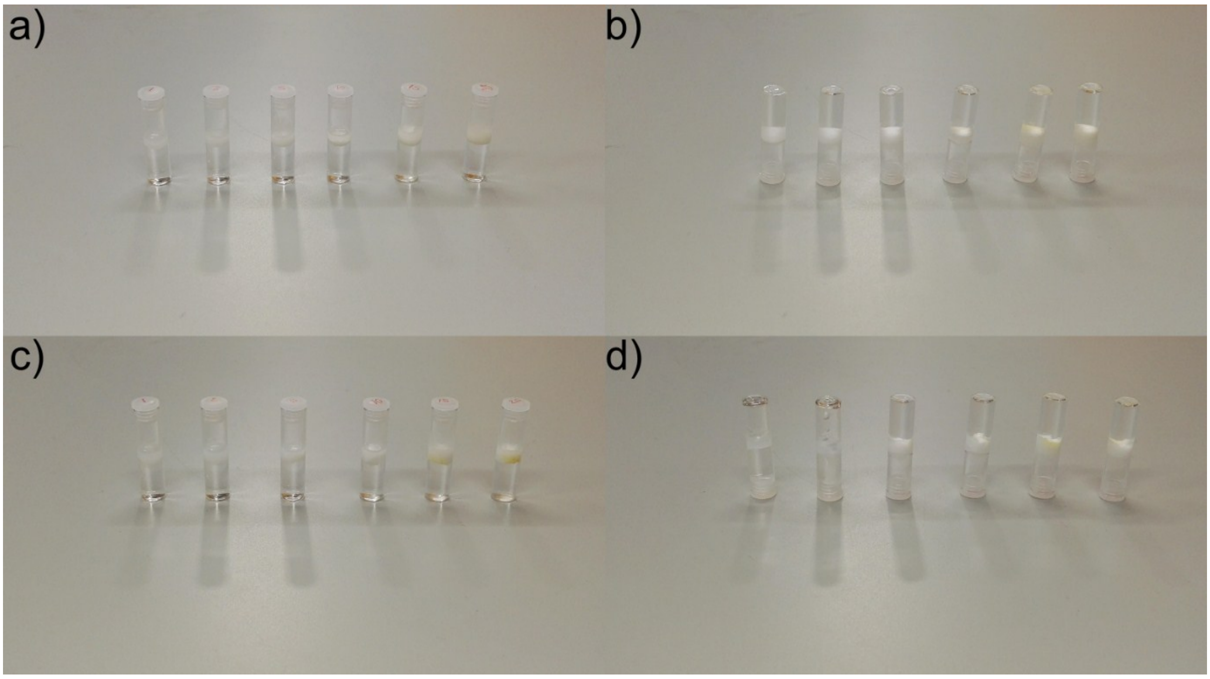

Figure S4: Photographs showing inversion tests for 1 mL of octadecene placed on 2 mL of deionised water. Gelator concentration was varied for both compound **1** (pictures a) and b)) and **2** (pictures c) and d)) to ensure final concentrations were: 1 wt. %, 2 wt. %, 5 wt. %, 10 wt. % and 20 wt. % from left to right.

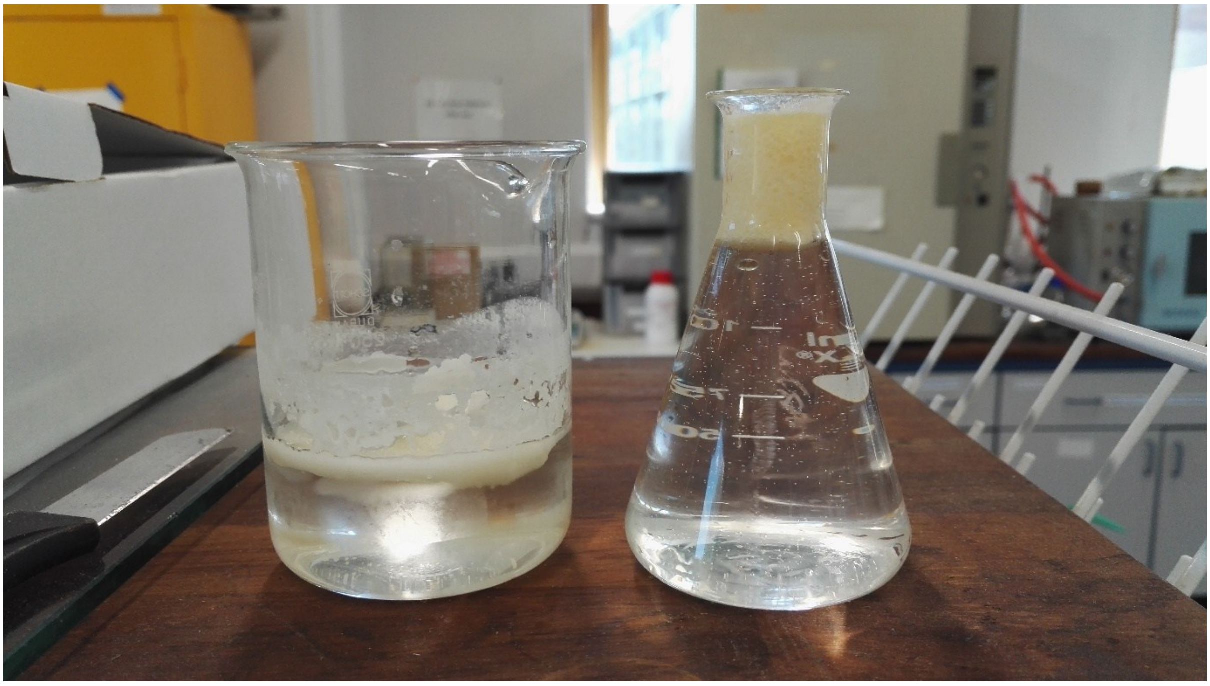

Figure S5: Photographs showing Compound **1** (left) and **2** (right) gelling 1-octadecene slicks on river water.

Table S1: Gelation tests were carried out at 4 wt% w/v LMWO on 4 g of selected oils placed on 4 ml seawater, forming a thick slick. The sample was then placed in an ice-bath and cooled to -5 ºC before injection of LMWO precursors. The gelation properties of the 2 compounds in oils of varying polarity. G indicates a gel formed (survived the inversion test) and the CGC estimated from inversion testing is given in brackets (units% w/v). NG indicates that no gel was formed, G* indicates that a gel was formed under the above conditions but did not survive the inversion test (with an estimate of CGC given in brackets) and D indicated that the LMWO precursors dissolved and did not gel.

Oil	Compound **1**	Compound **2**
Toluene	D	NG
Hexan-1-ol (Sigma Aldrich Ltd., reagent grade, 98%)	D	G* (10)
Kerosene (Alfa Aesar, low odor)	G* (10)[1]	NG
Mobil Super 3000 motor oil	G* (5)[1]	NG**
Vegetable oil (Tesco Ltd.)	G	NG
Octadec-1-ene (techn. grade, 90%, control, room temp)	G	G* (10)

[1] W. J. Peveler, H. Packman, S. Alexander, R. R. Chauhan, L. M. Hayes, T. J. Macdonald, J. K. Cockcroft, S. E. Rogers, D. G. A. L. Aarts, C. J. Carmalt, I. P. Parkin and J. C. Bear, *Soft Matter*, 2018, **14**, 8821–8827
** The 1-dodecylamine precursor froze before thorough mixing could occur. This was due to the highly viscous nature of the oil, highlighting the importance of rapid mixing, and the unsuitability of compound **2** for cold environments compared to compound **1**.

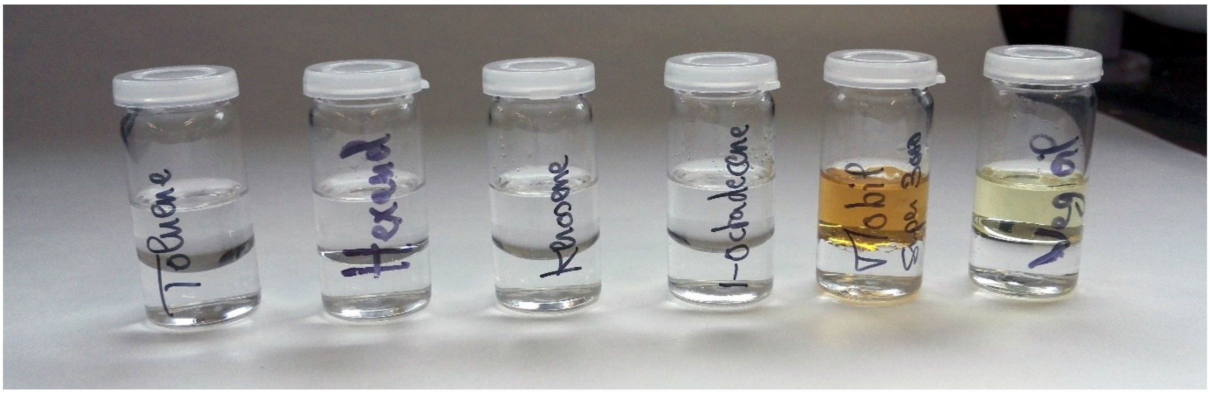

Figure S6: Photograph showing the different oil-on-water slicks described in the caption for Table S1, with toluene, hexan-1-ol, kerosene, Mobil Super 3000 motor oil, Vegetable oil and the octadec-1-ene control on seawater (Brighton, UK).

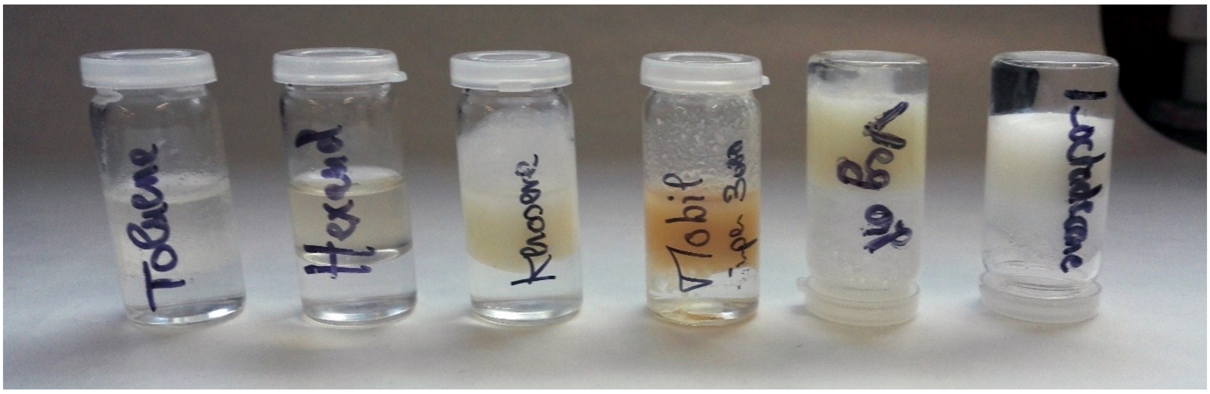

Figure S7: Photograph showing the gelation results for Compound **1** at 4wt% w/v with the vegetable oil (-5 °C) and 1-octadecene control (room temp) demonstrating successful inversion tests.

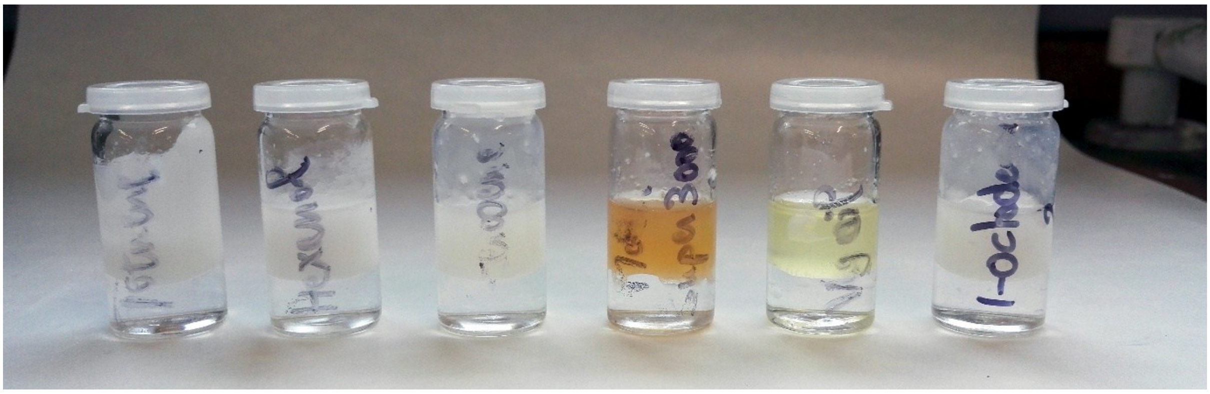

Figure S8: Photograph showing the gelation results for Compound **2** at 4wt% w/v. Results for Figures **S7** and **S8** are given in Table **S1**.

Part I: Preparation of 1 in situ and analysis of resulting gel

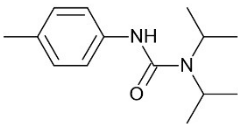

N'-(4-Methylphenyl)-*N,N*-dipropan-2-ylurea, compound **1**, made *in situ,* by co-adding from opposite ends of the vessel neat 1-isocyanato-4-methylbenzene (*p*-tolyl isocyanate) and neat *N*-(propan-2-yl)propan-2-amine (diisopropylamine) to the oil layer floating on top of a water layer. Gelation off the whole oil layer occurred within 30 seconds of the addition of the chemical precursors.

The NMR spectroscopy:

Instrumentation

The NMR spectra were acquired on a Bruker Avance III 400MHz (^{1}H) FT-NMR spectrometer equipped with a room temperature 5 mm probehead (PABBO BB-1H/D Z-GRD, broadband multinuclear, autotune) from Bruker BioSpin GmbH, Switzerland, and controlled with TopSpin 3.5.7 and Icon NMR 5.0.7 © 2017 Bruker Biospin GmbH. NMR data processing was performed using TopSpin 4.0.4 © 2018 Bruker Biospin GmbH.

Sample preparation

Samples (*ca.* 30 dry gelator or 100mg gelator + oil) were prepared in $CDCl_3$ (Goss Scientific Instruments Ltd., Crewe, UK) and spiked with a trace amount of tetramethylsilane (Merck, Gillingham, UK) as the 0 ppm internal reference for ^{1}H spectra.

NMR analysis

1D (^{1}H, ^{13}C DEPT-Q) and 2D (^{1}H-^{1}H COSY, ^{1}H-^{13}C HSQC) NMR experiments were carried out to confirm the presence of gelator **1**. The parameter sets used to acquire the datasets were the standard ones provided by the spectrometer manufacturer. The data were processed using TopSpin 3.6.1 (Bruker BioSpin GmbH, Rheinstetten, Germany).

1D ^{1}H (base optimised): 16 scans, 64K complex data points, 8.2KHz spectral width. 1D proton data were fast Fourier transformed after applying a single exponential apodisation window (lb = 0.2Hz) and zero filling to 64K real data points. The 1D ^{1}H spectrum was automatically referenced to internal TMS, automatically phase-corrected with manual adjustment of the phase as required, and automatically baseline corrected (using polynomial degree 5 curve fitting). Subsequent 1D and 2D spectra were referenced using the corresponding TMS-referenced ^{1}H spectrum as per IUPAC recommendations.[1]

1D ^{13}C DEPT-Q (base optimised): 256 scans, 64K complex data points, 24KHz spectral width. 1D carbon data were fast Fourier transformed after applying a single exponential apodisation window (lb = 1.0Hz) and zero filling to 64K real data points. The spectrum was automatically phase-corrected, with manual adjustment of the phase as required, and automatically baseline corrected (using polynomial degree 5 curve fitting).

2D ^{1}H-^{13}C HSQC (hsqcedetgpsp.3): 2 scans, 1024 x 256 complex data points, spectral width in F1 16.6KHz, in F2 3.0KHz (optimised from 1H). The data were fast Fourier transformed after applying quadratic sine apodisation windows in F2 (lb = 1.0Hz, GB =0, SSB = 2) and in F1 (lb = 0.3Hz, GB =0.1, SSB = 2) followed by zero filling to 1024 x 1024 real data points. The spectrum was automatically phase-corrected with manual adjustment of the phase as required, and automatically baseline corrected (using polynomial degree 5 curve fitting).

2D ^{1}H-^{1}H COSY (cosygpmfqf): 2 scans, 2048 x 128 complex data points, spectral widths 3.0KHz (optimised from 1H). The data were fast Fourier transformed after applying sine apodisation windows in F2 (lb = 1.0Hz, GB = 0, SSB = 0) and in F1 (lb = 0.3Hz, GB = 0.1, SSB = 0) followed by zero filling to 1024 x 1024 real data points. The spectrum was automatically baseline corrected (using polynomial degree 5 curve fitting).

NMR Results

Gelator 1 alone:
^{1}H NMR (400 MHz, $CDCl_3$) δ 7.25(2H, d, *J* = 8.0 Hz, 2'- and 6'-H), 7.08 (2H, d, *J* = 8.0 Hz, 3'- and 5'-H), 6.13 (1H, s, N**H**), 3.97 (2H, sept, *J* = 6.9 Hz, 2x **CH**(CH_3)$_2$), 2.28 (3H, s 4'-CH_3), 1.31 (12H, d, *J* = 6.9 Hz, 2x CH(**CH_3**)$_2$); ^{13}C NMR (100 MHz, $CDCl_3$) δ 154.78 (C=O), 136.75 (C_Q), 132.07 (C_Q), 129.32 (3' and 5'-CH), 119.84 (2' and 6'-CH), 45.38 (**CH**(CH_3)$_2$), 21.54 (CH(**CH_3**)$_2$), 20.70 (4'-CH_3).

Gelator 1 in the presence of oil (i.e. 1 formed *in situ*):
^{1}H NMR (400 MHz, $CDCl_3$) δ 7.25(2H, d, *J* = 8.1 Hz, 2'- and 6'-H), 7.07 (2H, d, *J* = 8.1 Hz, 3'- and 5'-H), 6.10 (1H, s, N**H**), 3.97 (2H, sept, *J* = 6.9 Hz, 2x **CH**(CH_3)$_2$),), 2.28 (3H, s 4'-CH_3), 1.32 (12H, d, *J* = 6.9 Hz, 2x CH(**CH_3**)$_2$); ^{13}C NMR (100 MHz, $CDCl_3$) δ 154.80 (C=O), 136.73 (C_Q), 132.03 (C_Q), 129.29 (5-CH), 119.80 (6-CH), 45.36 (**CH**(CH_3)$_2$), 21.50 (CH(**CH_3**)$_2$), 20.68 (4'-CH_3).

From the NMR spectrum of gelator **1** formed in the oil, it can be estimated that in the gel, the molar ratio oil-gelator **1** is 22:1.

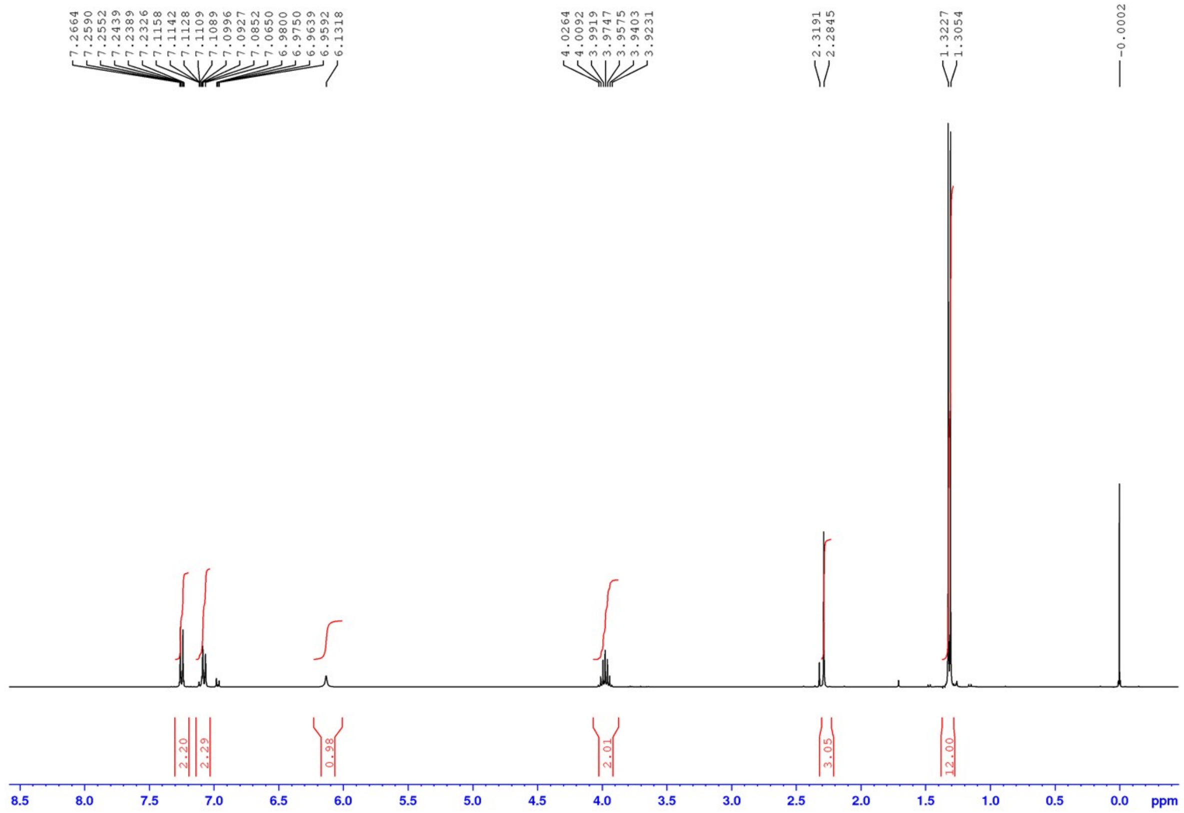

*Figure 1: ^{1}H NMR spectrum ($CDCl_3$) of gelator **1** made by solvent-free reaction of diisopropylamine with p-tolyl isocyanate.*

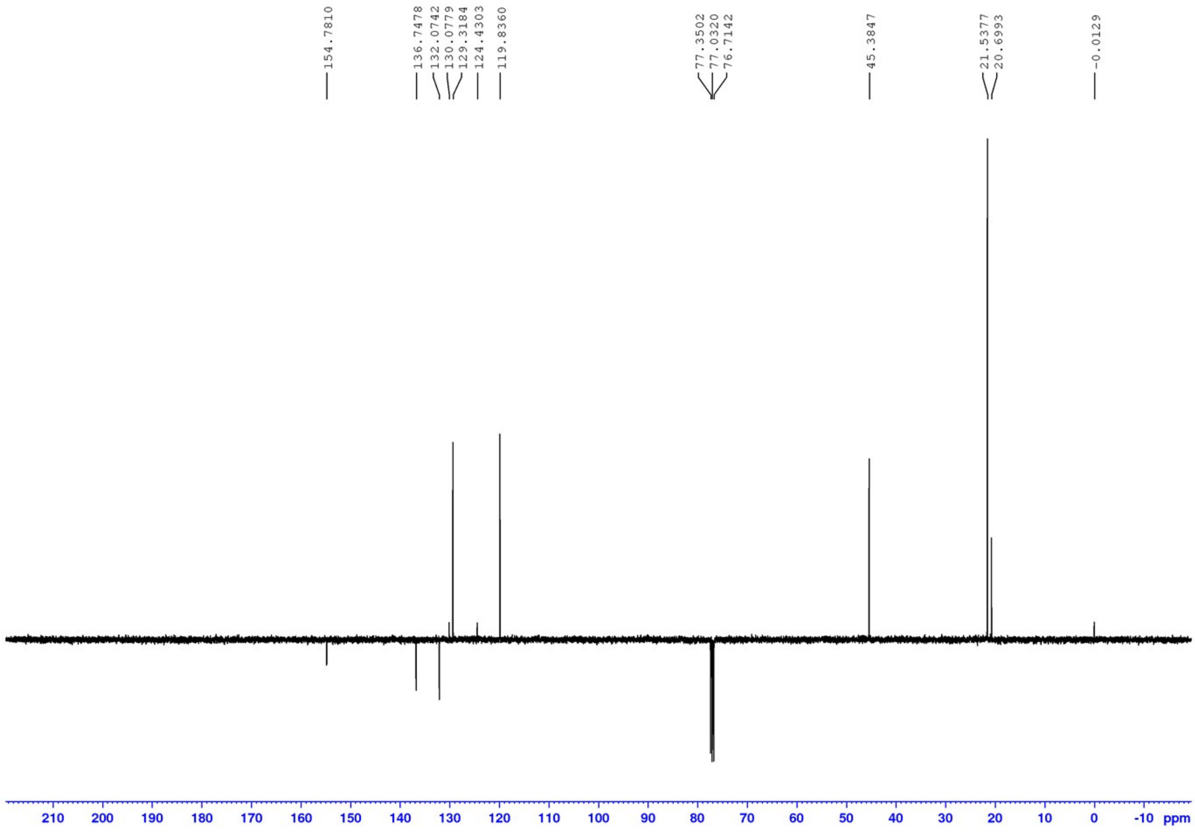

*Figure 2: ^{13}C DEPT-Q spectrum (CH and CH_3 +ve, CH_2 and C_Q -ve) ($CDCl_3$) of gelator **1** made by solvent-free reaction of diisopropylamine with p-tolyl isocyanate.*

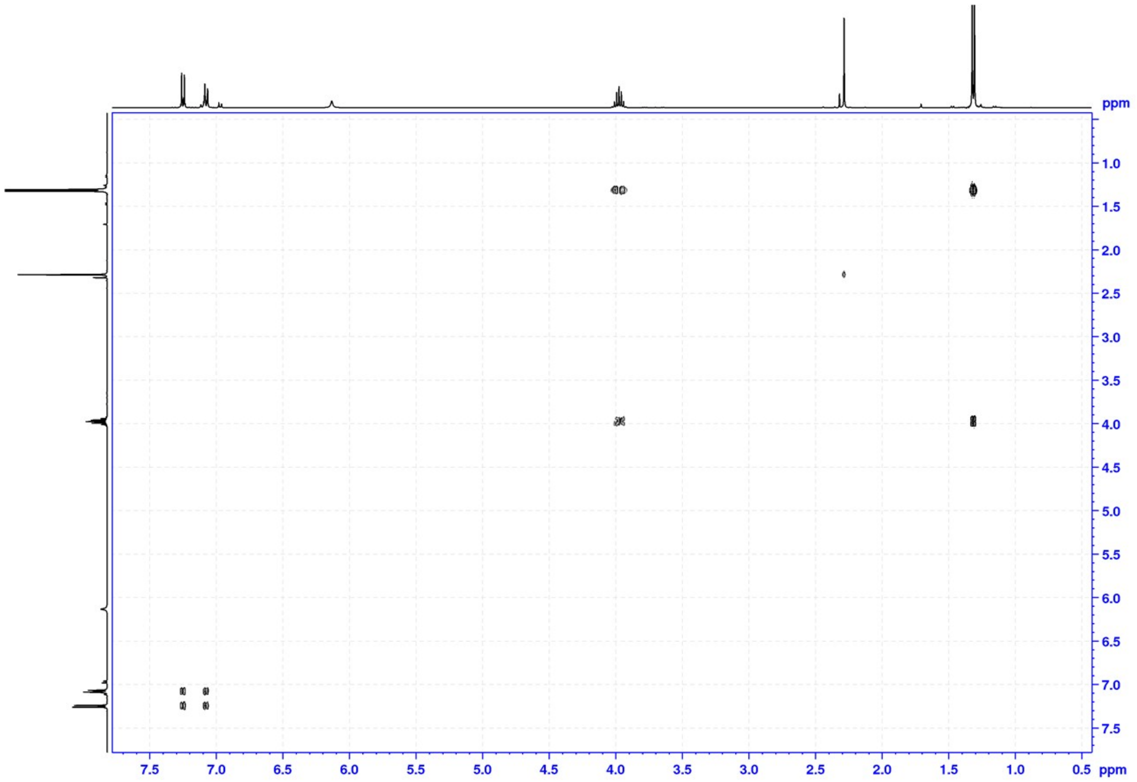

*Figure 3: ^{1}H-^{1}H COSY ($CDCl_3$) of gelator **1** made by solvent-free reaction of diisopropylamine with p-tolyl isocyanate.*

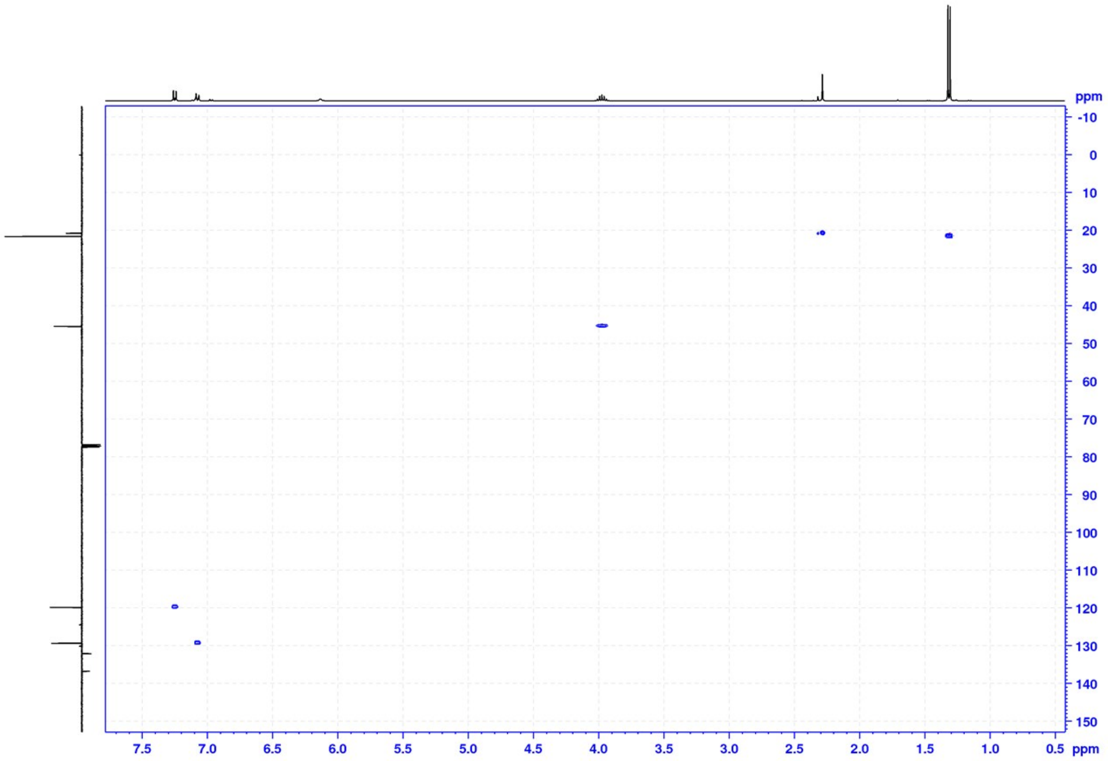

*Figure 4: ^{1}H-^{13}C HSQC ($CDCl_3$) of gelator **1** made by solvent-free reaction of diisopropylamine with p-tolyl isocyanate.*

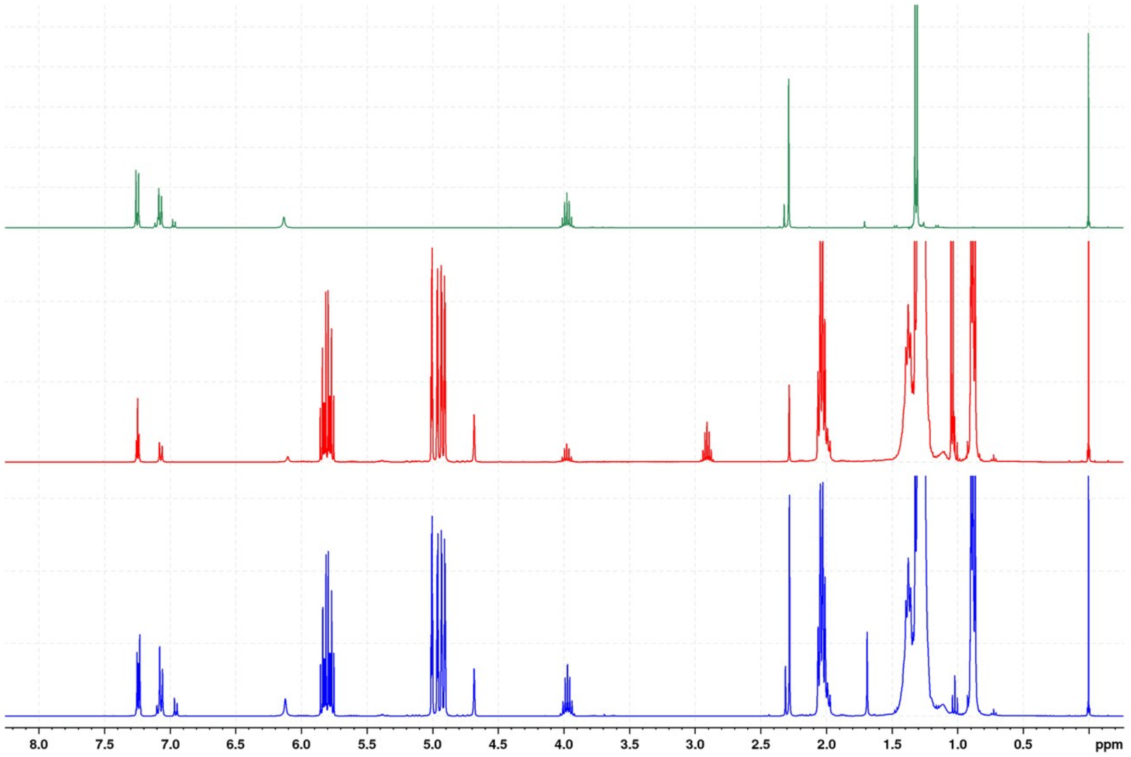

*Figure 5: ^{1}H NMR spectra ($CDCl_3$) of gelator-**1** made in-situ (2 attempts) vs neat*

Green = gelator **1** made by solvent-free reaction of diisopropylamine with *p-tolyl isocyanate*. Red = gelator **1** made by addition of diisopropylamine to *p-tolyl isocyanate* mixed with oil layer floating on water layer. Blue = gelator **1** made by co-addition of diisopropylamine and *p-tolyl isocyanate* into oil layer floating on water layer.

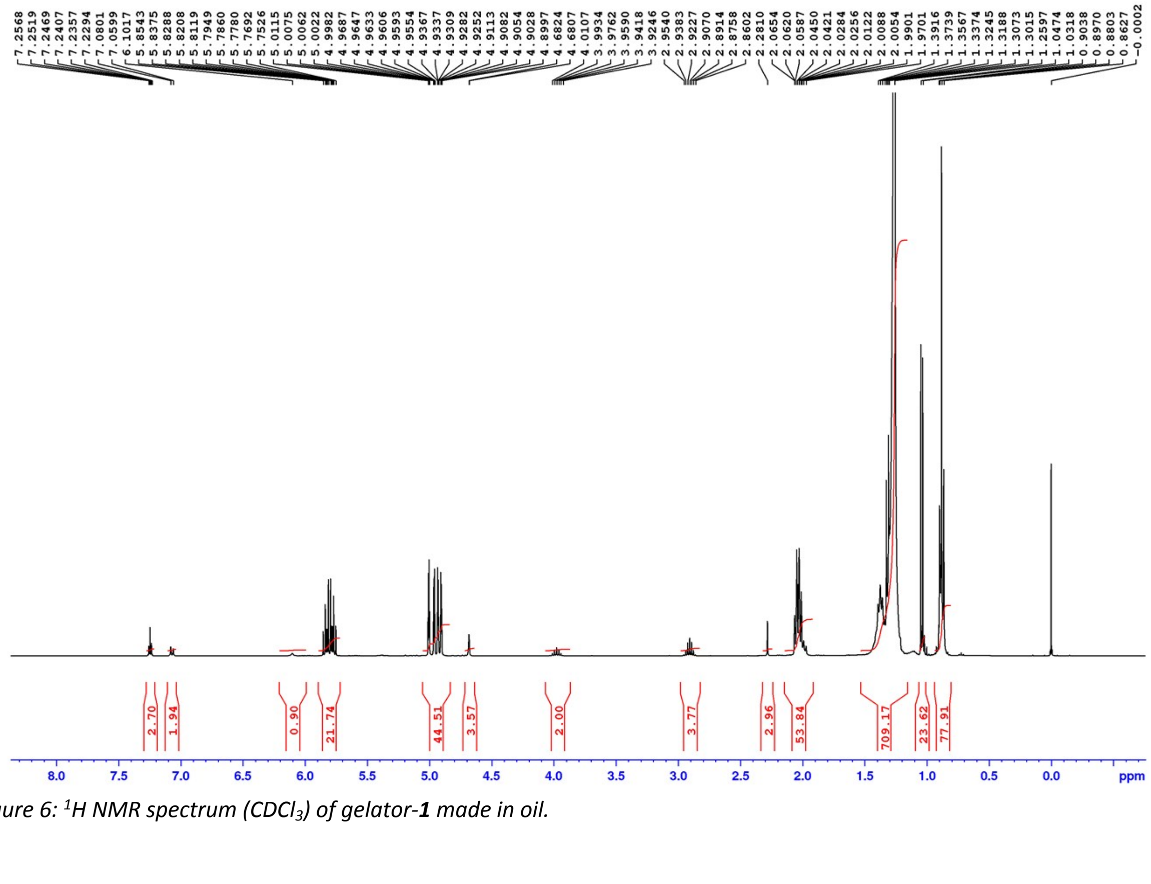

*Figure 6: ^{1}H NMR spectrum ($CDCl_3$) of gelator-**1** made in oil.*

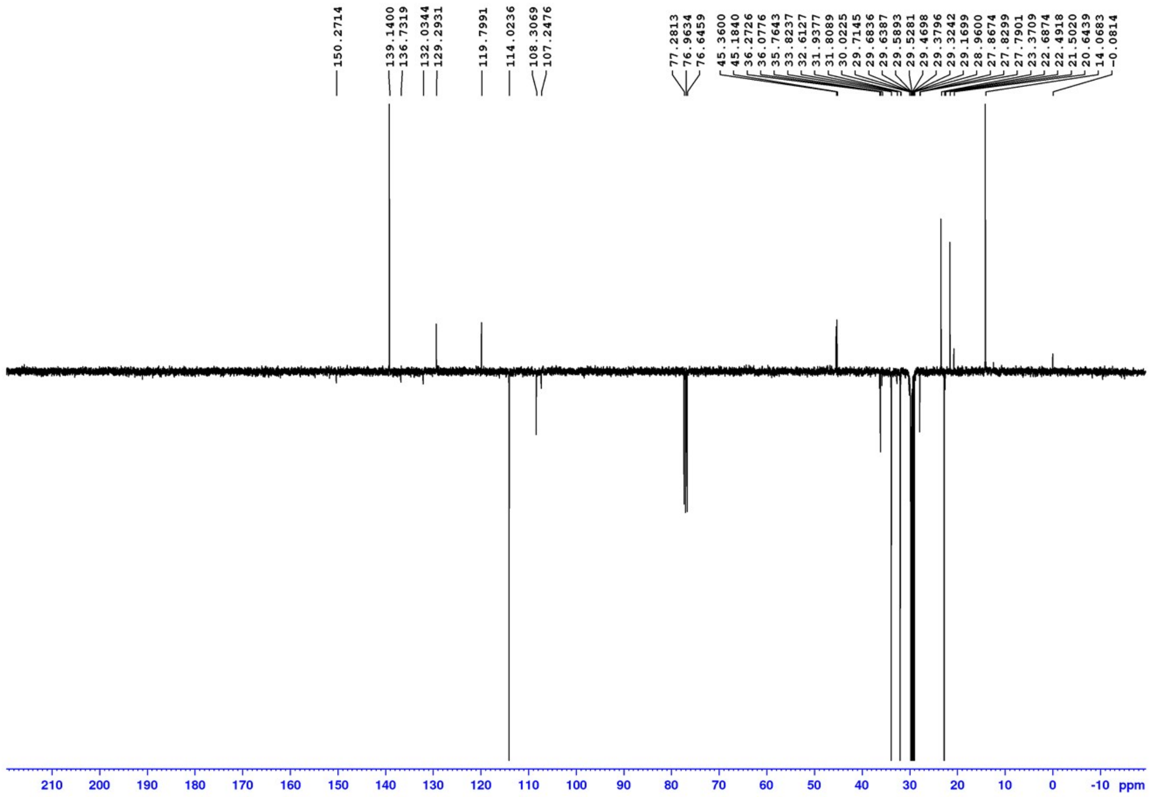

*Figure 7: ^{13}C DEPT-Q spectrum (CH and CH_3 +ve, CH_2 and C_Q -ve) ($CDCl_3$) of gelator-**1** made in oil.*

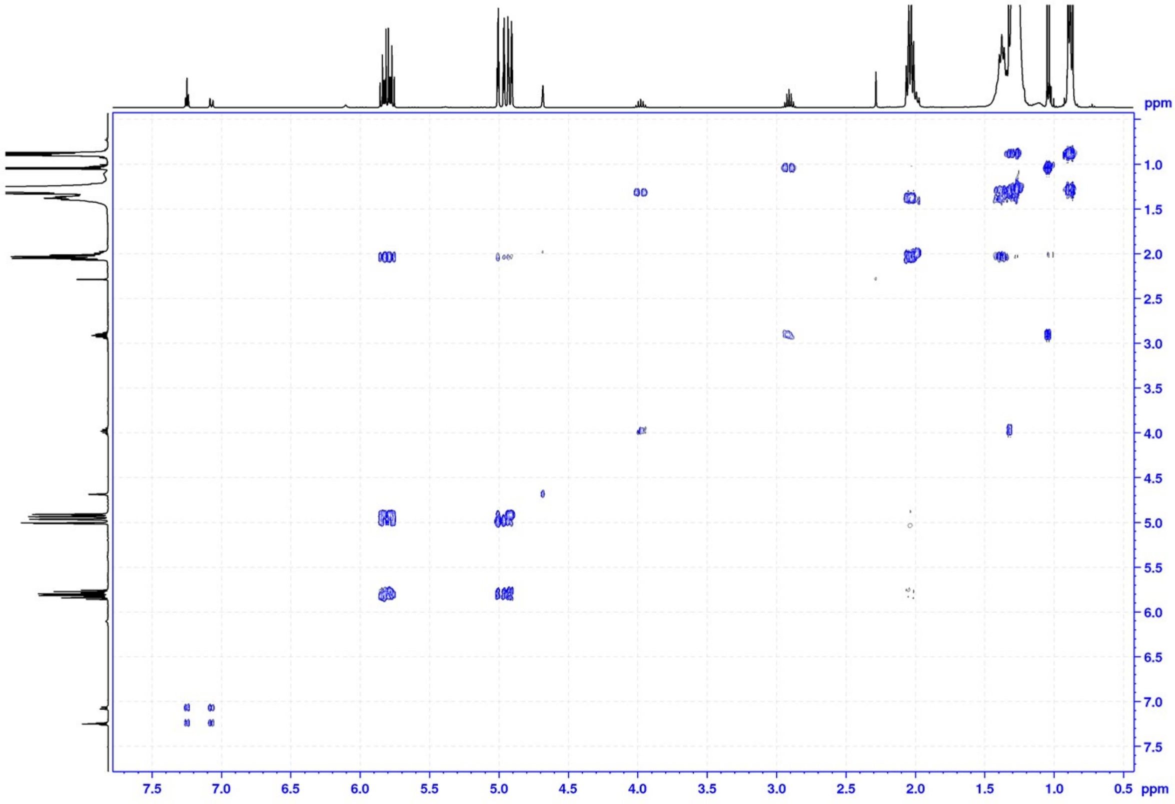

*Figure 8: ^1H-^1H COSY ($CDCl_3$) of gelator-**1** made in oil.*

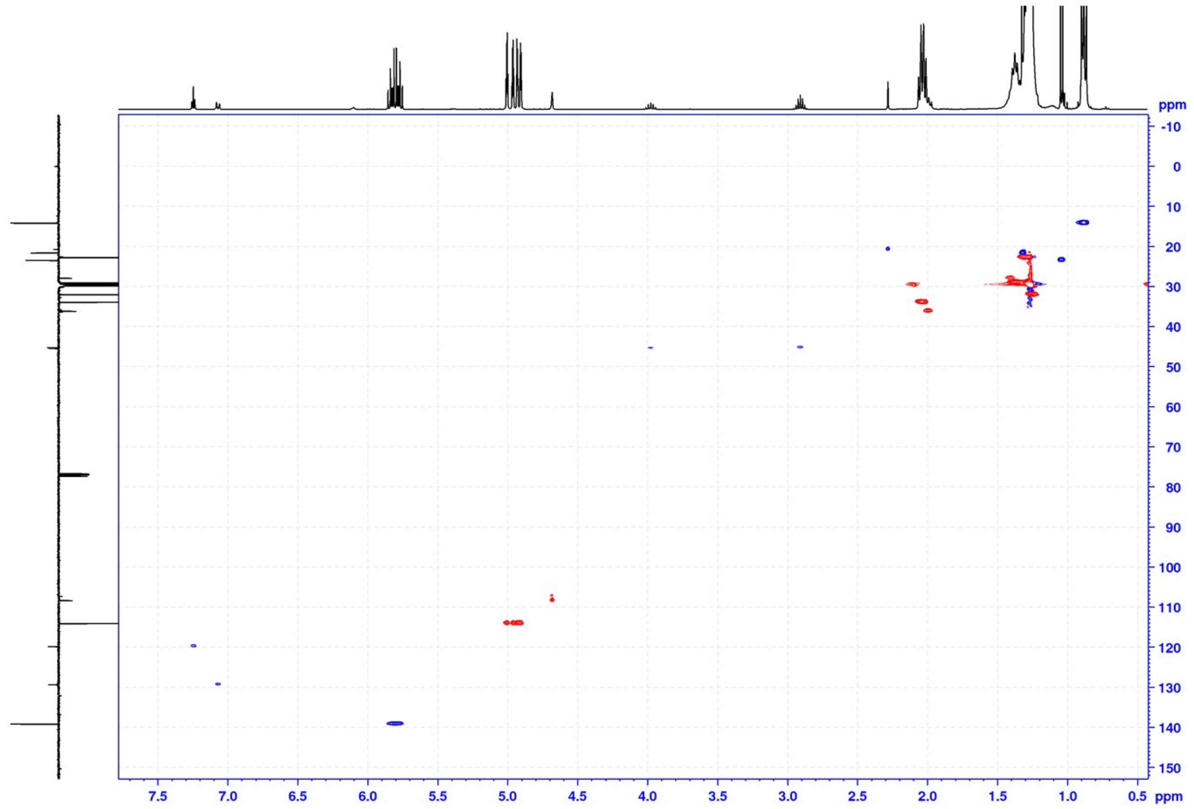

*Figure 9: ^{1}H-^{13}C HSQC ($CDCl_3$) of gelator-**1** made in oil.*

Part II: Preparation and analysis of 2

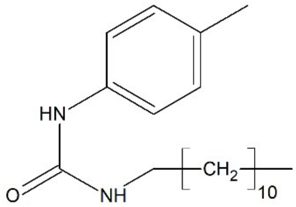

N-dodecyl-*N'*-(4-methylphenyl)-urea, compound **2**, made *in situ,* by adding neat 1-isocyanato-4-methylbenzene (*p*-tolyl isocyanate) to the n-hexane on top of a water layer followed by neat dodecan-1-amine (dodecylamine). Gelation of the whole organic layer occurred within 30 seconds of the addition of the chemical precursors.

NMR spectroscopy:

Instrumentation

The NMR spectra were acquired on a Bruker Avance III 600MHz (^{1}H) FT-NMR spectrometer equipped with a room temperature 5 mm PATXI 1H/D Z-GRD, $^{1}H\{^{13}C, ^{15}N\}$ probehead (Bruker BioSpin GmbH, Switzerland), and controlled with TopSpin 3.5.7 and Icon NMR 5.0.7 © 2017 Bruker Biospin GmbH. NMR data processing was performed using TopSpin 3.6.1 or TopSpin 4.0.6 (Bruker BioSpin GmbH, Rheinstetten, Germany).

Sample preparation

Sample (*ca.* 10 dry gelator) was prepared in DMSO-d_6 (Fluorochem Ltd., Hadfield, UK).

NMR analysis

1D (^{1}H, ^{13}C DEPT-Q) and 2D (^{1}H-^{1}H COSY, ^{1}H-^{13}C HSQC) NMR experiments were carried out to characterise gelator **2**. The parameter sets used to acquire the datasets were the standard ones provided by the spectrometer manufacturer. The data were processed using TopSpin 3.6.1 (Bruker BioSpin GmbH, Rheinstetten, Germany).

1D ^{1}H (base optimised): 16 scans, 64K complex data points, 12KHz spectral width. 1D proton data were fast Fourier transformed after applying a single exponential apodisation window (lb = 0.2Hz) and zero filling to 64K real data points. The 1D ^{1}H spectrum was automatically phase-corrected with manual adjustment of the phase as required, and automatically baseline corrected (using polynomial degree 5 curve fitting). The ^{1}H spectrum was referenced to the solvent residual signal at 2.50ppm[2]. Subsequent spectra were indirectly referenced to the ^{1}H spectrum.

1D ^{13}C DEPT-Q (deptqgpsp.2, base-optimised): 256 scans, 64K complex data points, 36KHz spectral width. 1D carbon data were fast Fourier transformed after applying a single exponential apodisation window (lb = 1.0Hz) and zero filling to 64K real data points. The spectrum was automatically phase-corrected, with manual adjustment of the phase as required, and automatically baseline corrected (using polynomial degree 5 curve fitting).

2D ^{1}H-^{13}C HSQC (hsqcedetgpsp.3): 2 scans, 1024 x 256 complex data points, spectral width in F1 25KHz, in F2 5.4KHz (optimised from 1H). The data were fast Fourier transformed after applying quadratic sine apodisation windows in F2 (lb = 1.0Hz, GB =0, SSB = 2) and in F1 (lb = 0.3Hz, GB =0.1, SSB = 2) followed by zero filling to 1024 x 1024 real data points. The spectrum was automatically phase-corrected with manual adjustment of the phase as required, and automatically baseline corrected (using polynomial degree 5 curve fitting).

2D ^{1}H-^{1}H COSY (cosygpmfqf): 2 scans, 2048 x 128 complex data points, spectral widths 5.4KHz (optimised from ^{1}H). The data were fast Fourier transformed after applying sine apodisation windows in F2 (lb = 1.0Hz, GB = 0, SSB = 0) and in F1 (lb = 0.3Hz, GB = 0.1, SSB = 0) followed by zero filling to 1024 x 1024 real data points. The spectrum was automatically baseline corrected (using polynomial degree 5 curve fitting).

2D ^{1}H-^{15}N HSQC (hsqcetgpsi): 2 scans, 4096 x 128 complex data points, spectral width in F1 24.3KHz, in F2 5.4KHz (optimised from ^{1}H). The data were fast Fourier transformed after applying quadratic sine apodisation windows in F2 (lb = 1.0Hz, GB =0, SSB = 2) and in F1 (lb = 0.3Hz, GB =0.1, SSB = 2) followed by zero filling to 2048 x 1024 real data points. The spectrum was automatically phase-corrected with manual adjustment of the phase as required, and automatically baseline corrected (using polynomial degree 5 curve fitting).

^{1}H-^{13}C HMBC (hmbcetgpl3nd): 2 scans, 4096 x 128 complex data points, spectral width in F1 33.5KHz, in F2 5.4KHz (optimised from ^{1}H). The data were fast Fourier transformed after applying sine apodisation windows in F2 (lb = 0Hz, SSB = 4) and a quadratic sine in F1 (lb = 0Hz, SSB = 2) followed by zero filling to 2048 x 1024 real data points. The spectrum was automatically phase-corrected, and automatically baseline corrected (using polynomial

degree 5 curve fitting). Finally the spectrum was displayed in magnitude mode (F2 magnitude calculation) to improve signal intensity.

NMR of gelator 2 after removal of n-hexane under vacuum:

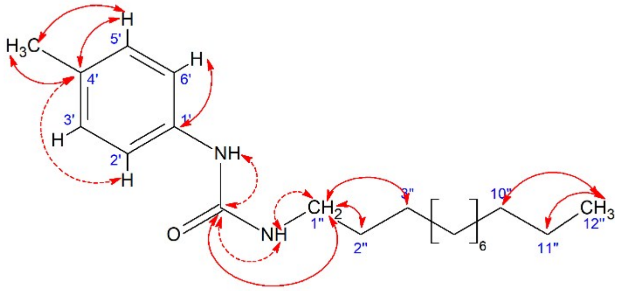

Figure 13: ^{1}H-^{13}C HMBC correlation red curly arrows: full = strong, dotted = weak. All labelled carbons were assigned iteratively using ^{1}H-^{1}H COSY, ^{1}H-^{13}C HSQC and ^{1}H-^{13}C HMBC except for 10' and 11' which cannot be unambiguously assigned.

^{1}H NMR (600 MHz, DMSO-d$_6$) δ 8.19 (1H, s, Ph**NH**CONHCH$_2$-) 7.24 (2H, d, J = 8.3 Hz, 2'- and 6'-H), 7.00 (2H, d, J = 8.3 Hz, 3'- and 5'-H), 6.00 (1H, t, J = 5.6 Hz, PhNHCO**NH**CH$_2$-), 3.05 (2H, brd dd, J = 12.8, 6.8 Hz, 1''-**CH$_2$**), 2.21 (3H, s 4'-CH$_3$), 1.42-1.38 (2H, m, 2''-**CH$_2$**), 1.29-1.21 (18H, m, **-(CH$_2$)$_9$**CH$_3$)$_,$0.85 (3H, t, J = 7.0 Hz, 12''-CH$_3$)

^{13}C NMR (150 MHz, DMSO-d$_6$) δ 155.73 (C=O), 138.52 (1'-C$_Q$), 130.02 (4'-C$_Q$), 129.43 (3'- and 5'-CH), 118.16 (2'- and 6'-CH), 39.49 (1''-CH$_2$), 31.74 (CH$_2$), 30.24 (2''-CH$_2$), 29.49 (CH$_2$), 29.48 (CH$_2$), 29.46 (CH$_2$), 29.45 (CH$_2$), 29.23 (CH$_2$), 29.15 (CH$_2$), 26.84 (3''-CH$_2$), 22.53 (CH$_2$), 20.73 (4'-CH$_3$), 14.38 (12''-CH$_3$)

^{15}N NMR (60.8MHz, DMSO-d$_6$) 103.60 (Ph**NH**CONHCH$_2$-), 86.20 (PhNHCO**NH**CH$_2$-) (from projection of f1 of ^{1}H-^{15}N HSQC).

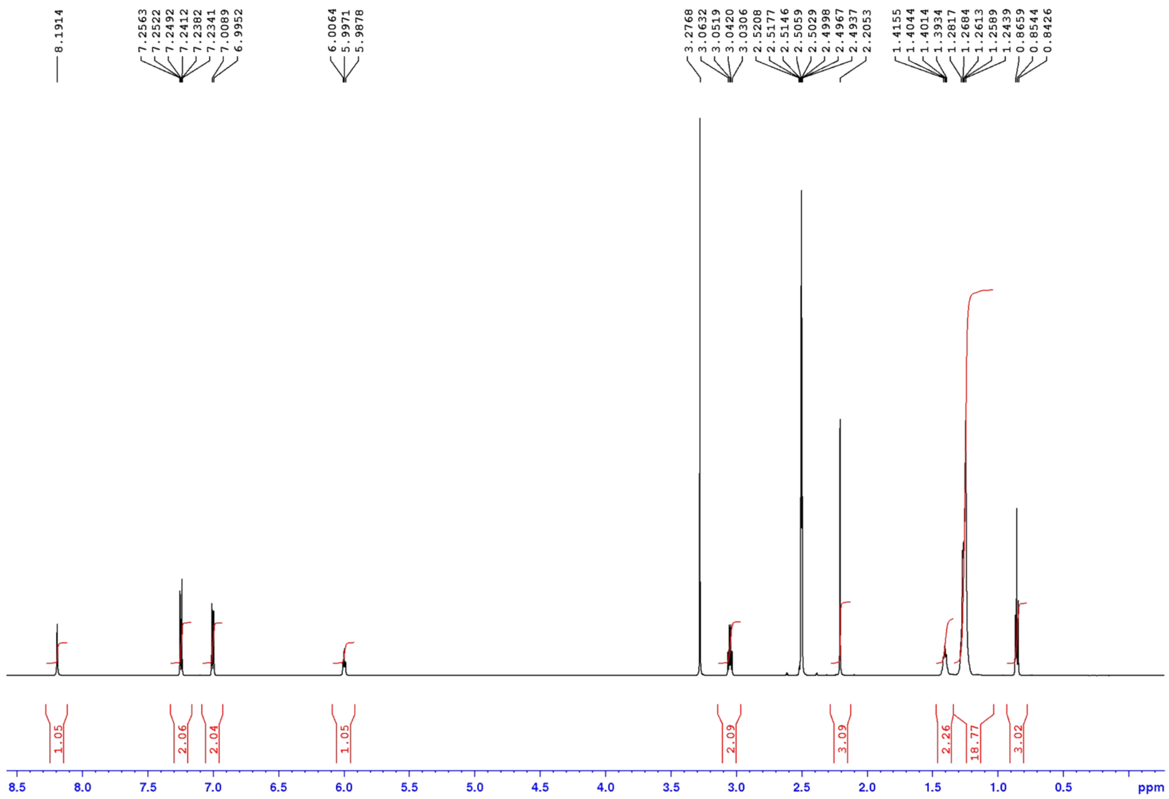

*Figure 14: ^{1}H NMR spectrum of gelator **2** (DMSO-d_6)*

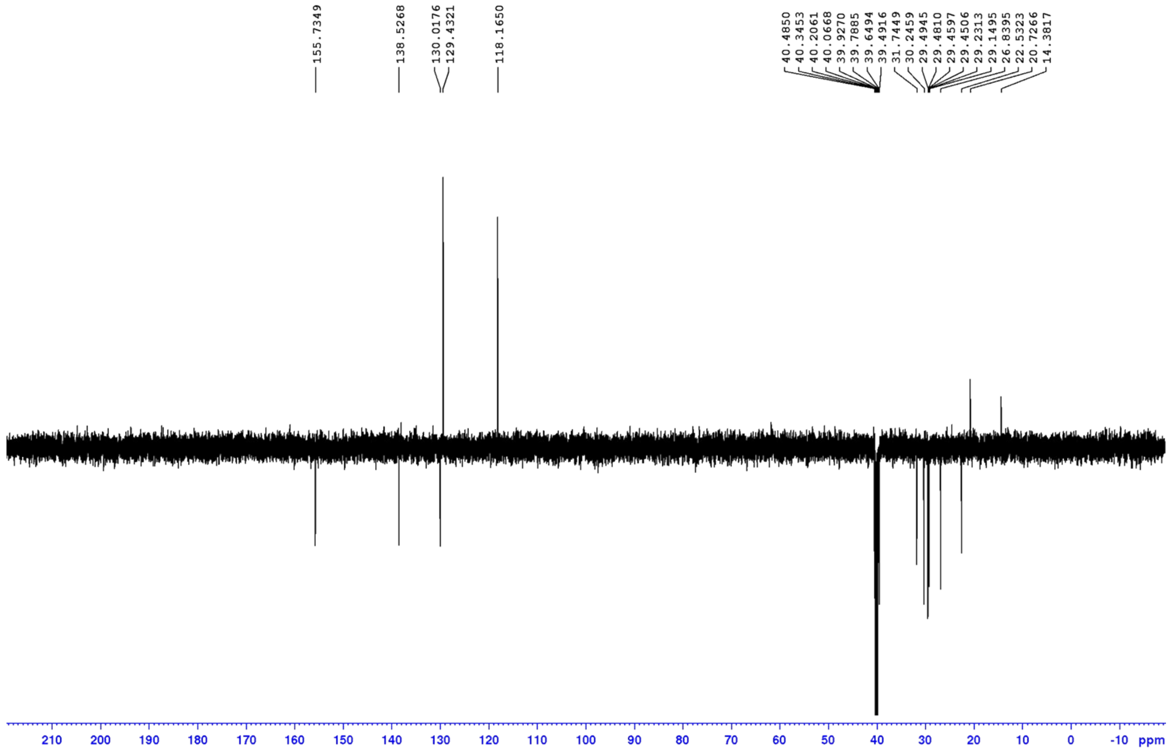

*Figure 15: ^{13}C DEPT-Q spectrum (CH and CH_3 +ve, CH_2 and CQ -ve) of gelator **2** (DMSO-d_6)*

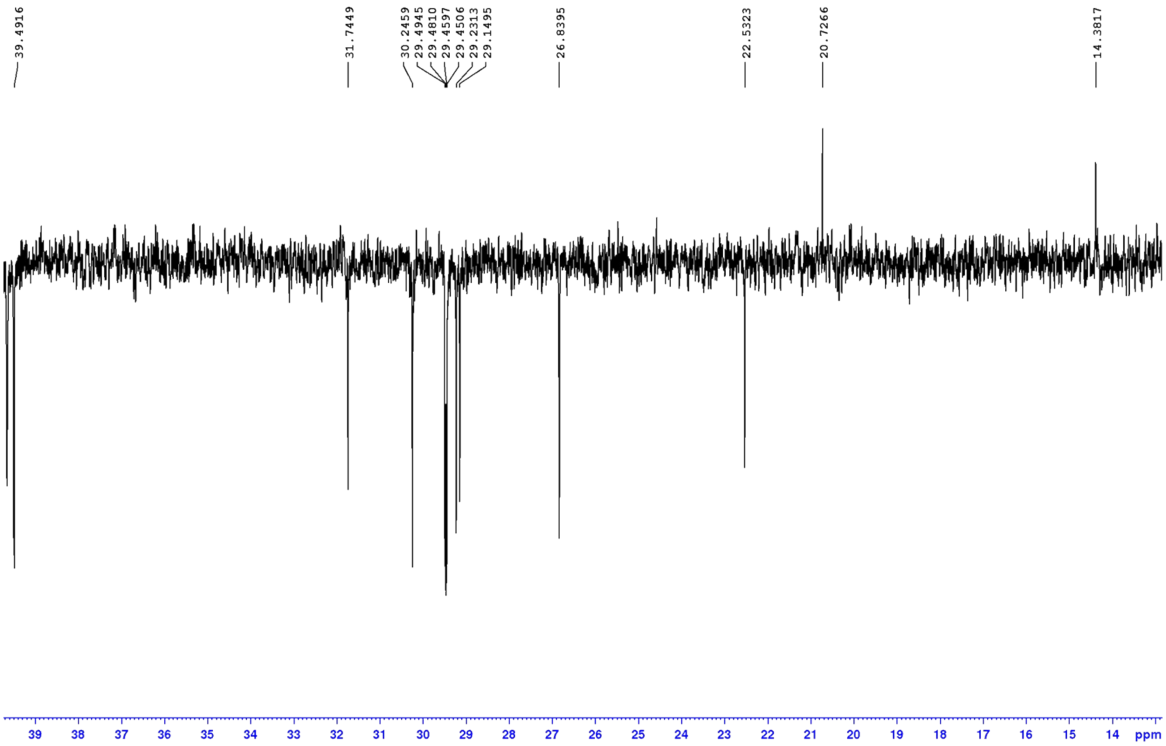

*Figure 16: Expansion of ^{13}C DEPT-Q spectrum of gelator **2** (DMSO-d_6), showing 11 CH_2 peaks and 2 CH_3 peaks*

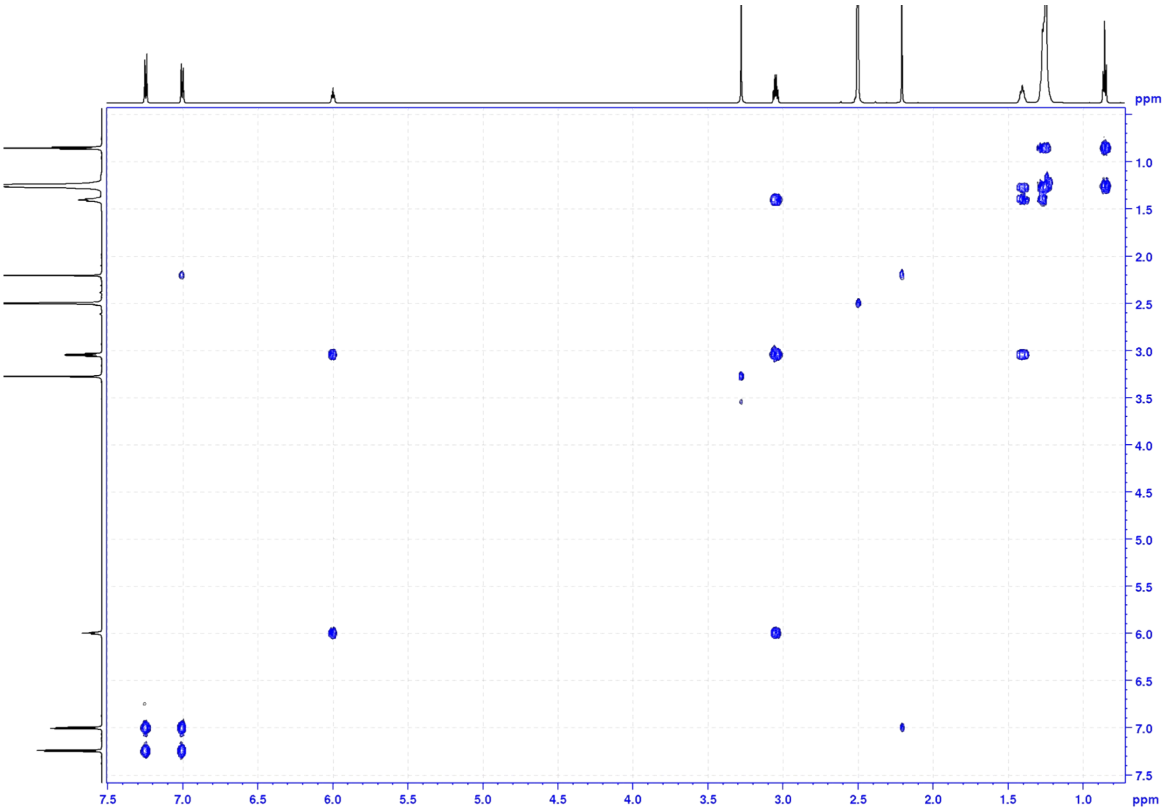

*Figure 17: ^{1}H-^{1}H COSY of gelator **2** (DMSO-d_6)*

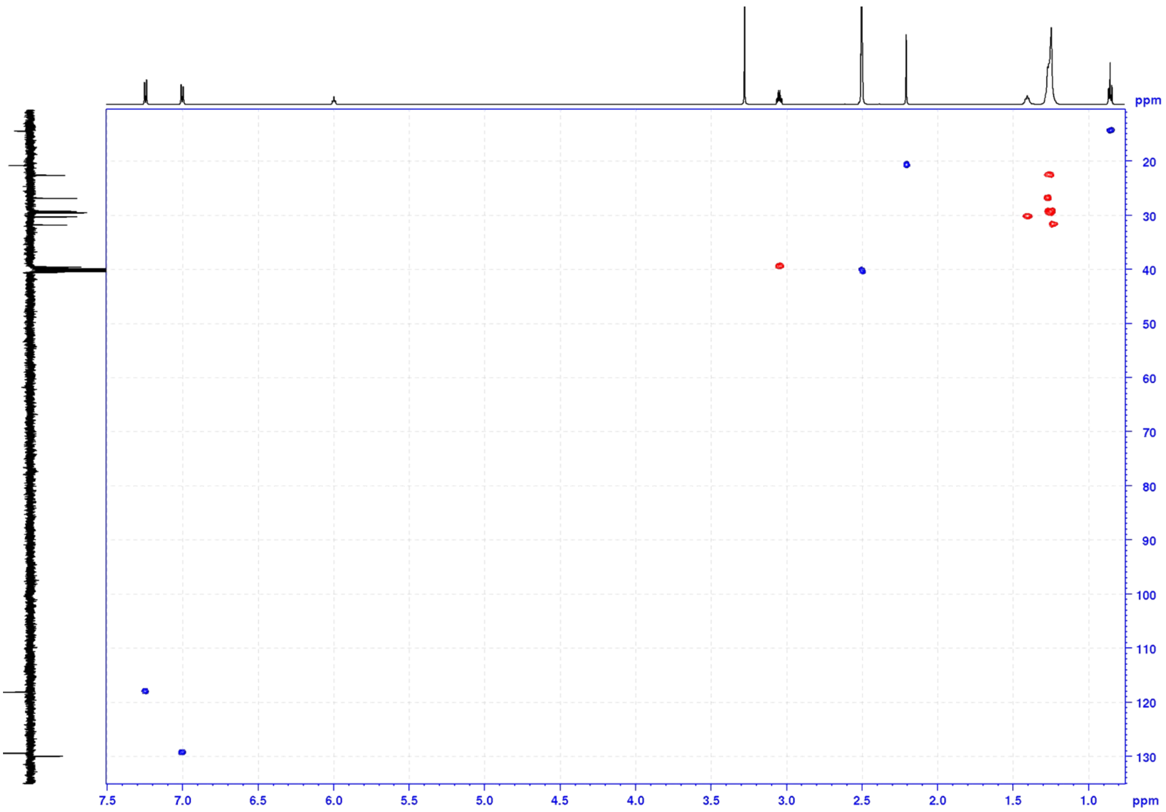

*Figure 18: ^{1}H-^{13}C HSQC of gelator **2** (DMSO-d$_6$)*

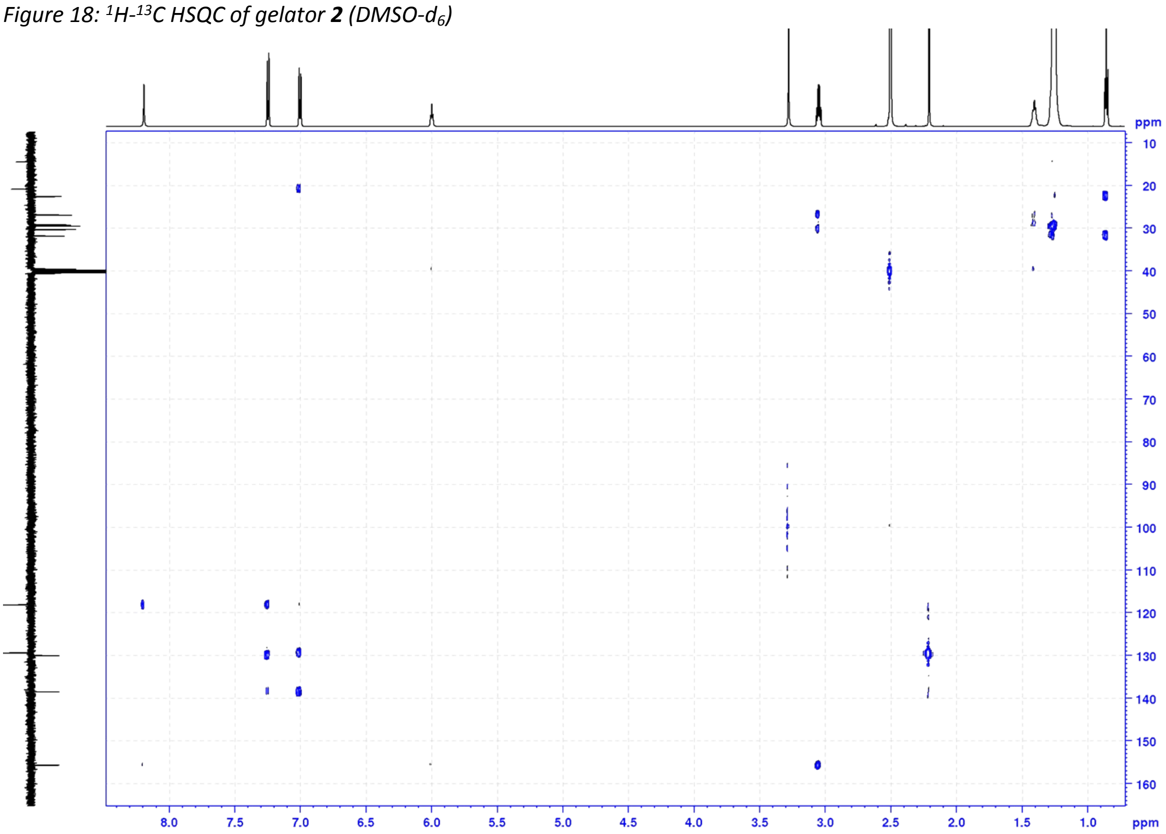

*Figure 19: ^{1}H-^{13}C HMBC of gelator **2** (DMSO-d$_6$)*

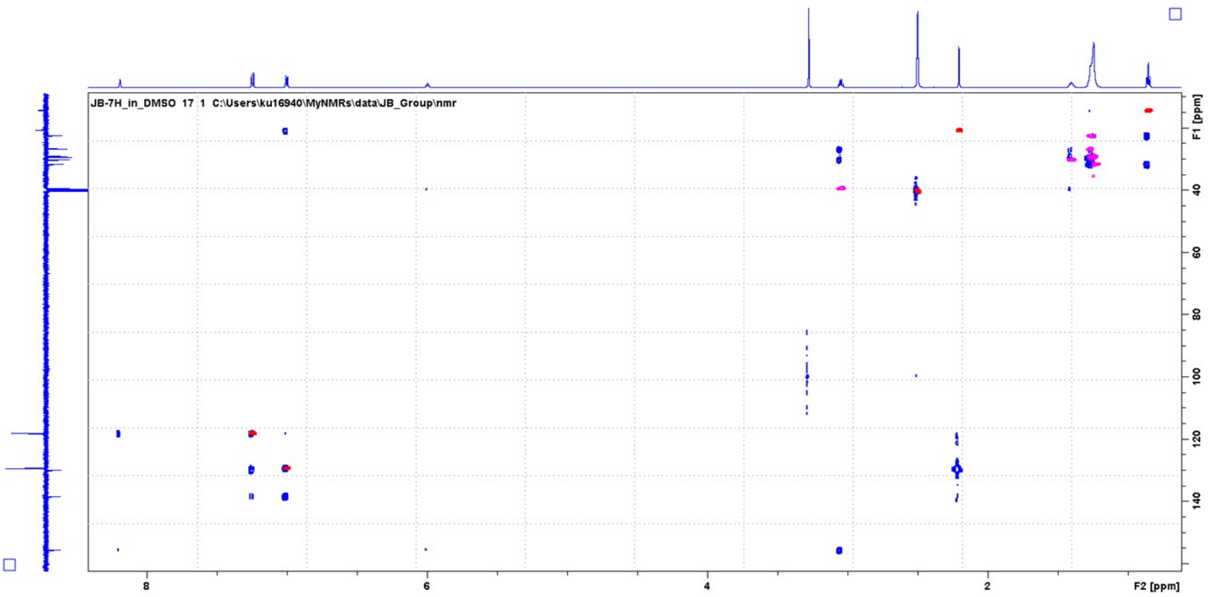

*Figure 20: ^1H-^{13}C HSQC (red for CH and CH_3, pink for CH_2) overlayed on ^1H-^{13}C HMBC (blue) of gelator **2** (DMSO-d_6)*

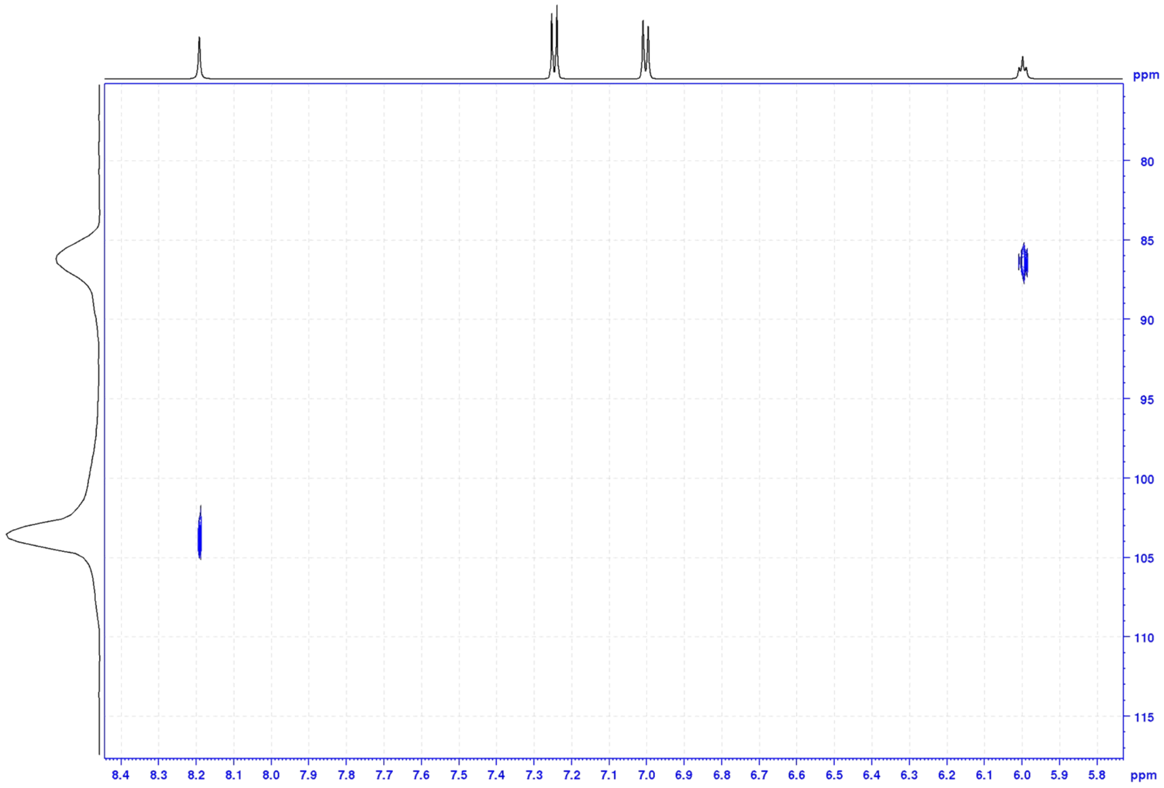

*Figure 21: ^1H-^{15}N HSQC of gelator **2** (DMSO-d_6)*

Part III: Aqueous leaching experiments

Instrumentation

The NMR spectra were acquired on a Bruker Avance III 600MHz (^1H) FT-NMR spectrometer equipped with a room temperature 5 mm PATXI 1H/D Z-GRD, $^1H\{^{13}C, ^{15}N\}$ probehead (Bruker BioSpin GmbH, Switzerland), and controlled with TopSpin 3.5.7 and Icon NMR 5.0.7 © 2017 Bruker Biospin GmbH.

Sample preparation

Sea/river water (10 ml) was placed in a vial and 1-octadecene (1 ml) was layered on top of it. The precursors to the gelator were co-added to the organic phase to in order produce a gel with a 10 wt% gelator:oil ratio. After approximately 15 minutes, the aqueous layer below the newly formed gel was gently homogenised and sampled for NMR analysis. Each NMR tube was assembled using the aqueous layer from the gelation experiment (0.540 ml), D_2O (0.060 ml) and a bolus (0.030 ml) of dimethyl sulfone (Merck, Gillingham, UK) stock solution (9.10 mg in 1.000 ml) as internal standard. Prior to NMR analysis, samples were spiked with a trace amount of 3-(Trimethylsilyl)propionic acid-d4 sodium salt (TSP) (Merck, Gillingham, UK) as the internal reference (0 ppm).

NMR analysis

1D ^1H spectra were acquired with water suppression using the manufacturer supplied pulse program noesygppr1d, which uses presaturation during relaxation and mixing time. The water peak position and optimal pulse length were automatically adjusted for each sample which was maintained at a constant 298° K during the measurements. Relevant acquisition parameters included a receiver gain (RG) set to 128, 64 scans, 64K complex data points and 12.3 KHz spectral width. The acquisition time was 2.65 seconds and the relaxation delay (D1) was 30 seconds to allow relaxation of the protons between pulses. The quantitation standard dimethyl sulfone was chosen as it is fully soluble in water and its well resolved singlet did not overlap with the peaks of interest. These conditions were selected to achieve quantitative integrals and yield appropriate signal to noise ratios for the analytes of interest.[3,4]

The data were processed using TopSpin 3.6.1 (Bruker BioSpin GmbH, Rheinstetten, Germany) applying fast Fourier transform to the data after applying a single exponential apodisation window (lb 1.0Hz, to minimise noise) and zero filling to 64K real data points. The spectra were automatically referenced to internal TSP, automatically phase-corrected with manual adjustment of the phase as required. The baseline was corrected automatically using polynomial degree 5 curve fitting, prior to manual integration of the peaks of interest. In order to unequivocally match aromatic signals to the individual components detected in the mixture, water-suppressed 2D COSY spectra were acquired using the manufacturer's supplied pulse program "cosygpprqf". Relevant parameters were 16 scans, 4096 x 256 complex data points and a spectral width of 8.4KHz (optimised from ^1H). The centre of the spectrum was obtained from the ^1H spectrum and used as the water suppression frequency. Processing involved sine window functions F2 (lb = 1.0Hz, GB = 0, SSB = 0) and in F1 (lb = 0.3Hz, GB = 0.1, SSB = 0) and zero filling in F1 to produce a 2048 * 2048 real data points.

The ^1H NMR spectra obtained are presented staked together on **Figure 23**. NMR signals that were looked for in the leaching experiments are given in **Table 1**. The quantitation results are given in **Table 2**.

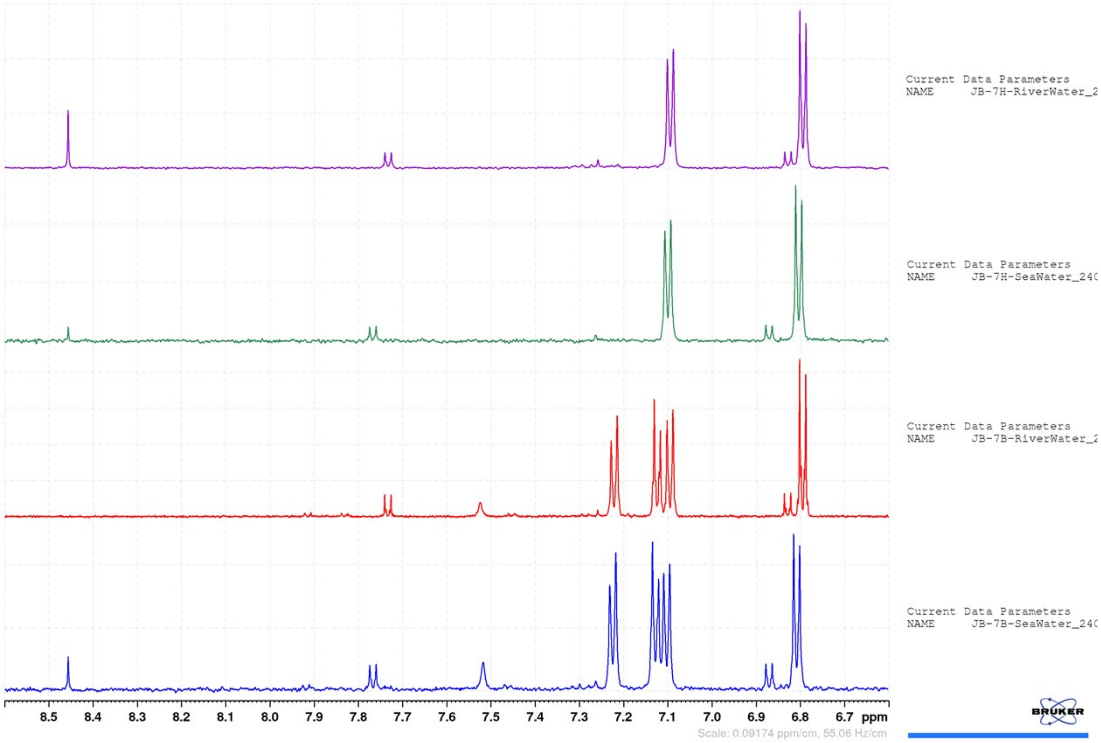

Figure 23: Stacked spectra of aqueous layer from leaching experiments

Table 1: NMR signals for reagents vs NMR signals of pure gelators

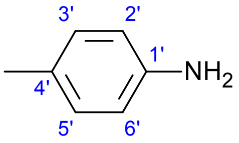

***p*-Tolylamine**

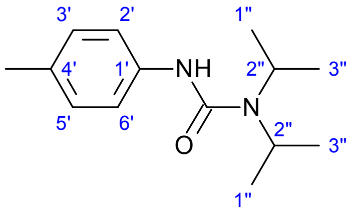

Gelator 1

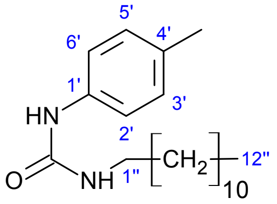

Gelator 2

***p*-Tolylamine (H_2O/D_2O 9:1, TSP)**

ID	Shift	multiplicity	integral
2' & 6'	7.12	d, 8.4Hz	2H
3' & 5'	6.85	d, 8.4Hz	2H
4'-CH_3	2.25	s	3H
**Impurity***			
2' & 6'	7.76	d, 8.Hz	2H
3' & 5'	6.86	d, 8.7Hz	2H

Diisopropylamine (D_2O, TSP)

2''	2.95	sept., 6.4Hz	2H
1'' & 3''	1.03	d, 6.4Hz	12H

Dodecylamine (D_2O, TSP)

1''	2.95	Brd d, 7.7 Hz	2H
2''	1.63	m	2H

Gelator 1 ($CDCl_3$, TMS)

ID	Shift	multiplicity	integral
2' & 6'	7.25	d, 8.0Hz	2H
3' & 5'	7.08	d, 8.0Hz	2H
4'-CH_3	2.28	s	3H
NH	6.13	s	1H
2''	3.97	sept., 6.9Hz	2H
1'' & 3''	1.31	d, 6.9Hz	12H

Gelator 2 (DMSO-D_6, TMS)

2' & 6'	7.24	d, 8.3Hz	2H
3' & 5'	7.00	d, 8.3Hz	2H
4'-CH_3	2.21	s	3H
NH	8.19	s	1H
1''	3.05	dd, 12.8 and 6.8 Hz	2H
2''	1.38	m	2H

**3'' – 11''**	1.39-1.24	m	18H	**3'' – 11''**	1.29-1.21	m	18H
**12''**	0.87	t, 7.0Hz	3H	**12''**	0.85	t, 7.0Hz	3H

* Impurity found in some batches of commercial p-Tolyl isocyanate.

Table 2: Amounts of leachates found in water leaching experiments (ppm)

Leaching experiment	*p*-Tolylamine	Diisopropylamine	Dodecylamine	Gelator
**1 on sea water**	30	27 (S/N = 58)	-	50
**1 on river water**	35	28	-	61
**1 2% on river water****	33	51	-	40
**2 on sea water**	26	-	ND	ND
**2 on river water**	27	-	ND	ND

Internal standard was dimethylsulfone, δ_H (D_2O) = 3.15 ppm (internal reference TSP)
ND = not detectable above noise level, ** sample from 2% wt/wt gelator:oil ratio (i.e. 2 tenth of previous sample).
The values reported above are those determined from a single NMR sample on one peak for each analyte where a signal to noise ratio above 240 was measured (unless otherwise noted). When other peaks were available for integration which had S/N in as low as 77 the resulting concentrations were essentially the same as those presented above. The results for **2** on river water were determined on an aged sample. Aging seemed to have little effect on quantitation results for **2** (data in spreadsheet provided with this supporting information).

References:

[1] R. K. Harris, E. D. Becker, S. M. Cabral de Menezes, P. Granger, R. E. Hoffman, K. W. Zilm, *Pure Appl. Chem.* **2008**, *80*, 59–84.
[2] H. E. Gottlieb, V. Kotlyar, A. Nudelman, *J. Org. Chem.* **1997**, *62*, 7512–7515.
[3] G. F. Pauli, B. U. Jaki, D. C. Lankin, *J. Nat. Prod.* **2007**, *70*, 589–595.
[4] S. K. Bharti, R. Roy, *Trends Anal. Chem.* **2012**, *35*, 5–26.
